# Xenopax for the treatment of steroid-refractory acute graft-versus-host disease: the RELAX study

**DOI:** 10.1186/s40779-025-00640-0

**Published:** 2025-09-29

**Authors:** Le-Qing Cao, Wen-Xuan Huo, Er-Lie Jiang, Yue-Wen Fu, Xiao-Jun Xu, Ping-Chong Lei, Ming-Feng Zhao, Zhi Chen, Shu-Xia Guo, Xiao-Bing Huang, Yan-Ming Zhang, Xian-Jing Wang, Guan-Chen Bai, Feng-Bo Jin, Qing-Sheng Li, Ming-Yang Deng, Hao Zhang, Xin-Feng Wang, Xiao-Jun Huang, Xiao-Dong Mo

**Affiliations:** 1 Peking University People’s Hospital, Peking University Institute of Hematology, National Clinical Research Center for Hematologic Disease, Beijing Key Laboratory of Cell and Gene Therapy for Hematologic Malignancies, Peking University, Beijing, 100044 China; 2 State Key Laboratory of Experimental Hematology, National Clinical Research Center for Blood Diseases, Haihe Laboratory of Cell Ecosystem, Institute of Hematology and Blood Diseases Hospital, Chinese Academy of Medical Sciences and Peking Union Medical College, Tianjin, 300020 China; 3 Department of Hematology, Henan Cancer Hospital, Affiliated Cancer Hospital of Zhengzhou University, Zhengzhou, 450008 China; 4 Children’s Hospital, Zhejiang University School of Medicine and National Clinical Research Center for Child Health, Hangzhou, 310052 China; 5 Department of Hematology, Henan Provincial People’s Hospital, Zhengzhou, 450003 China; 6 Department of Hematology, Tianjin First Central Hospital, Tianjin, 300192 China; 7 Department of Hematology, Wuhan Children’s Hospital (Wuhan Maternal and Child Healthcare Hospital), Tongji Medical College, Huazhong University of Science and Technology, Wuhan, 430016 China; 8 Department of Hematology, People’s Hospital of Zhengzhou, Zhengzhou, 450003 China; 9 Department of Hematology, Sichuan Provincial People’s Hospital, Affiliated Hospital of University of Electronic Science and Technology of China, Chengdu, 610072 China; 10 Department of Hematology, Huai’an Second People’s Hospital, Huai’an, 223002 Jiangsu China; 11 Department of Hematology, the Third People’s Hospital of Zhengzhou, Zhengzhou, 450003 China; 12 Department of Hematology, the Affiliated Tai’an City Central Hospital of Qingdao University, Tai’an, 271000 Shandong China; 13 Anhui Public Health Clinical Center, the First Affiliated Hospital of Anhui Medical University, Hefei, 230022 China; 14 The First Affiliated Hospital of Anhui Medical University, Hefei, 230022 China; 15 Department of Hematology, the Second Xiangya Hospital, Central South University, Changsha, 410011 China; 16 Department of Hematology, Affiliated Hospital of Jining Medical University, Jining, 272067 Shandong China; 17 Department of Hematology, Affiliated Hospital of Nantong University, Nantong, 226001 Jiangsu China; 18 Peking-Tsinghua Center for Life Sciences, Academy for Advanced Interdisciplinary Studies, Peking University, Beijing, 100871 China

**Keywords:** Xenopax, Acute graft-versus-host disease (aGVHD), Steroid refractory (SR), Allogeneic hematopoietic stem cell transplantation (allo-HSCT)

## Abstract

**Background:**

Steroid-refractory (SR) acute graft-versus-host disease (aGVHD) is the major cause of early mortality after allogeneic hematopoietic stem cell transplantation (allo-HSCT). Xenopax, a novel and the only available humanized interleukin-2 (IL-2) receptor antagonist, has been approved as a category 2 biological product by the National Medical Products Administration. This study aims to evaluate the efficacy, safety, and prognostic factors of xenopax treatment for SR-aGVHD in real-world settings.

**Methods:**

This was a multicenter, retrospective analysis that included SR-aGVHD patients who received xenopax at 17 hospitals across China. The data were collected from the electronic medical records in transplant databases. The primary endpoint was the 28-day overall response rate (ORR), encompassing both partial and complete responses. This study also included independent historical SR-aGVHD cohorts treated with best available treatments (BATs, *n* = 1009) as controls.

**Results:**

In total, 172 SR-aGVHD patients were included in this study. Xenopax was administered either as monotherapy (*n* = 60) or in combination with other second-line treatments (*n* = 112). The ORR was 64.5% [95% confidence interval (CI) 57.3–71.7%] on day 28 and 82.6% (95% CI 76.9–88.3%) at any time after xenopax treatment. The 2-year probabilities of disease-free survival, overall survival, non-relapse mortality (NRM), and relapse after xenopax treatment were 57.0% (95% CI 49.9–65.0%), 68.0% (95% CI 61.4–75.4%), 24.2% (95% CI 18.0–30.9%), and 19.0% (95% CI 12.8–25.2%), respectively. The ORR and survival were similar between patients with and without prior second-line treatments. The conditioning regimen and human leukocyte antigen disparity did not impact the efficacy of xenopax treatment. According to the multivariate analysis, the presence of grade III–IV aGVHD did not adversely affect the therapeutic response or survival. Xenopax also showed some superiority over BATs in historical cohorts.

**Conclusions:**

Our real-world findings suggest that xenopax is an effective and safe treatment for SR-aGVHD.

**Supplementary Information:**

The online version contains supplementary material available at 10.1186/s40779-025-00640-0.

## Background

Allogeneic hematopoietic stem cell transplantation (allo-HSCT) is a curative therapy for most hematological malignancies and nonmalignant hematological disorders [1–4]. Acute graft-versus-host disease (aGVHD) is an important post-transplantation complication, with severe cases being a major cause of early transplant-related mortality [5, 6]. Corticosteroids remain the first-line therapy for aGVHD, but their associated overall response rate (ORR) is approximately 50% [7], and the outcomes of steroid-refractory (SR)-aGVHD patients are poor [8].

Interleukin-2 (IL-2), the key factor in the pathogenesis of aGVHD, can activate donor cytotoxic T lymphocytes, which makes IL-2 receptor (IL-2R) antagonists a crucial treatment option for SR-aGVHD [9–12]. Several studies have documented the effectiveness of IL-2R antagonists in the treatment of SR-aGVHD [13–16]. A large-scale real-world study revealed that the cumulative ORR on day 28 was 79.4% for SR-aGVHD patients receiving basiliximab (Simulect; Novartis Pharma AG, Basel, Switzerland) [17], a chimeric mouse-human IL-2R monoclonal antibody. A recent meta-analysis further reported a superior ORR at any time after treatment with basiliximab (81%) compared with treatment with inolimomab (54%) and denileukin diftitox (56%). Additionally, the complete response rates (CRRs) for basiliximab (55%) were also better than those for inolimomab (30%) and denileukin diftitox (37%) [18].

Unlike basiliximab, xenopax (a recombinant humanized anti-CD25 monoclonal antibody) is a humanized IL-2R antagonist featuring a high human sequence content (90%) (Additional file 1: Table S1) and has been approved as a category 2 therapeutic biologic by the National Medical Products Administration (NMPA) in China. Following the global withdrawal of daclizumab (formerly marketed as Zenapax^®^ and Zinbryta^®^), xenopax is currently the only humanized IL-2R antagonist approved in China [19]. In a single-center study involving 64 SR-aGVHD patients, the ORR was 83% (CRR 58%), and the overall survival (OS) and non-relapse mortality (NRM) were 72.9% and 25.9%, respectively [20]. However, several limitations of this study are notable, such as the low proportion of patients with multiorgan involvement, the high prevalence (70%) of human leukocyte antigen-matched sibling donor transplants, the unclear impact of prior second-line therapies on the outcomes, and the insufficient identification of prognostic variables associated with xenopax treatment. Thus, the efficacy and safety of xenopax should be further evaluated in SR-aGVHD patients.

Although aGVHD is one of the most common complications after allo-HSCT, it remains an orphan disease [21], which limits larger-scale randomized controlled trials (RCTs). In addition, the clinical situations of SR-aGVHD patients are very complicated, and the strict inclusion and exclusion criteria of RCTs would negatively impact the generalizability of the results. Thus, real-world studies are close to clinical practice and could provide valuable guidance for the treatment of SR-aGVHD [22].

Here, a multicenter cohort study (ReaL-worLd study for Acute GVHD with Xenopax, RELAX study) to examine the safety, efficacy, and prognostic variables of xenopax in patients with SR-aGVHD was conducted. This investigation represents a real-world study of xenopax treatment for SR-aGVHD, providing comprehensive insights into its clinical performance.

## Methods

### Study design

A multicenter, retrospective study involving patients with SR-aGVHD who received xenopax treatment at 17 hospitals across China between January 1, 2020 and October 31, 2023 was conducted, with the last follow-up conducted on September 30, 2024 (details of the participating institutions and investigators are provided in the Additional file 1: List of investigators). Institutional review board approval for this research was obtained from each participating hospital (2023PHB429-001), and the study complied with the principles of the Declaration of Helsinki.

### Transplantation regimens

The major conditioning regimens and protocols for GVHD prophylaxis were consistent with the recommendations of the consensus from the Chinese Society of Hematology [23]. Briefly, the major GVHD prophylactic protocol included a calcineurin inhibitor, mycophenolate mofetil, and short-term methotrexate, and the patients who underwent haploidentical donor or unrelated donor HSCT also received antithymocyte globulin (ATG) for GVHD prophylaxis (Additional file 1: Table S2).

### Patients

Our source population included 172 patients treated with xenopax following allo-HSCT at the participating sites. Patients had to be diagnosed with grade II–IV SR-aGVHD and receive a minimum of 1 xenopax injection to be eligible for the study [24]. Steroid refractoriness or dependence was defined according to the criteria specified in international guidelines [25–27]. The exclusion criteria were as follows: (1) aGVHD caused by cellular therapies (e.g., donor lymphocyte infusion); (2) the presence of features indicative of chronic GVHD (cGVHD; e.g., overlap syndrome); and (3) incomplete medical records.

This study also included independent historical SR-aGVHD cohorts treated with best available treatments (BATs, *n* = 1009), including basiliximab (*n* = 940) [17], mesenchymal stem cells (MSCs) (*n* = 14) [28], ruxolitinib (*n* = 15) [29], and MSCs plus basiliximab (*n* = 40) [30] (Additional file 1: Table S3), to further compare the efficacy of xenopax with that of other second-line treatments. In addition, we also made an indirect comparison about ORR and survival between SR-aGVHD patients receiving xenopax and ruxolitinib in the REACH1 study [31].

### Data collection

The necessary data were collected from the electronic medical records of the transplant databases of each participating hospital (Additional file 1: Methods). Two experienced physicians specializing in allo-HSCT independently reviewed the collected data to ensure accuracy and consistency.

### GVHD treatment

Xenopax [Jiannipai; Sunshine Guojian Pharmaceutical (Shanghai) Co., Ltd.] was administered at a dose of 1 mg/kg on days 1, 4, and 8, followed by weekly doses until the aGVHD severity decreased to below grade II [20]. Steroid tapering was managed according to the clinical practices of each center.

Xenopax can be utilized in two primary ways: 1) As a monotherapy, xenopax can be administered alone, either as the initial treatment at the time of SR-aGVHD diagnosis or as the sole salvage therapy, replacing other systemic second-line treatments. 2) As a combined therapy, xenopax is administered concurrently with other systemic second-line therapies at the time of SR-aGVHD diagnosis or added to prior second-line treatments. The dose of steroids should be tapered gradually after the addition of xenopax; however, standard protocols for the decrease in the number of steroids were not available, which were mainly based on each center’s competence and experience. The protocols for the administration of other second-line treatments before or combined with xenopax are described in the Additional file 1: Methods.

### Endpoints and evaluations

The primary endpoint was the ORR on day 28, which included achieving a partial response (PR) or complete response (CR). Responses were evaluated based on the highest aGVHD grade and stage observed in each organ, with assessments conducted at least weekly after xenopax treatment (Additional file 1: Methods). Notably, responses were required to persist for at least 3 weeks without the initiation of other systemic treatments.

The secondary endpoints included the ORR at any time, cGVHD [32], OS, disease-free survival (DFS), NRM, and relapse of hematological malignancies (Additional file 1: Methods).

### Statistical analysis

The number of patients eligible for xenopax treatment was estimated based on the 28-day ORR of the BATs group in the REACH2 study [27]. The present study was planned to detect a 28-day ORR of 49% (i.e., a 10% increase) in patients receiving xenopax treatment from the reference rate of the REACH2 study of 39%, controlling for type I and II error rates at 5% and 20%, respectively. Considering an expulsion rate of 15%, a total of 172 patients were planned to be enrolled. Sample size calculation is described in the Additional file 1: Methods.

Patient characteristics and GVHD data were compared between groups using *χ*^2^ tests and Fisher’s exact tests for categorical data, and Student’s *t*-test for continuous data after confirming normal distribution (Shapiro–Wilk test) and homogeneity of variances (Levene’s test). Descriptive statistics were typically reported as the median (range) for continuous variables, or as *n* (%) for categorical variables. Proportional-hazards assumptions were verified by Schoenfeld residuals. Kaplan–Meier curves were generated to determine survival probabilities. The cumulative incidences of the therapeutic response, GVHD, mortality, and relapse were calculated with competing risks by the Fine–Gray sub-distribution hazard model [33]. Univariate Cox regressions followed by multivariable analyses were performed to evaluate the potential impacts of covariates on clinical outcomes. The univariate analysis was conducted using log-rank tests with an assumed type I error of 0.10, and the covariates included in the univariate analysis are listed in the Additional file 1: Methods. Cox proportional hazards regression models were applied for candidate variables with *P* < 0.10. *P* < 0.05 was considered statistically significant for other analyses (Additional file 1: Methods).

In sensitivity analyses, patients who received xenopax were propensity-matched to those who received BATs using the nearest-neighbor method and a 2% caliper with the following parameters: age, severity of aGVHD before second-line treatment, and refined Minnesota aGVHD risk scores before second-line treatment. Statistical tests were performed using SPSS v26 (SPSS Inc., IBM, Armonk, NY, USA), Power Analysis and Sample Size software (PASS 23.0.4), and R v3.6.2 (http://www.r-project.org).

A simplified cost-effectiveness analysis from the healthcare payer perspective that included 4 therapies for SR-aGVHD was conducted, namely, basiliximab, MSCs, ruxolitinib, and xenopax. The unit price of the drug was sourced from local healthcare pricing databases. Cost-effectiveness was estimated by dividing the total drug cost, which was calculated by multiplying the unit price with the median effective number of administrations per patient, by the treatment response rate. This approach provided a basic assessment of the economic efficiency of the drug in managing the condition, facilitating a preliminary comparison with alternative treatments.

## Results

### Demographics and clinical features of patients who received xenopax treatment

A total of 172 SR-aGVHD patients receiving xenopax were included in this study. The characteristics of patients and patients with aGVHD are presented in Table 1 and Additional file 1: Table S4. Before xenopax treatment, more than half of the patients (55.8%) had grade III–IV aGVHD, and more than 70% of the patients (74.4%, *n* = 128) had gut involvement. The median time from the aGVHD diagnosis to the initiation of xenopax treatment was 7 d (range, 3−54 d), and the median follow-up duration after treatment was 495 d (range, 3−1502 d). A total of 506 doses of xenopax were administered, and the median doses of xenopax for patients who achieved an ORR were 3 doses (range, 1–7 doses). The second-line treatments administered before xenopax treatment included mycophenolate mofetil alone (*n* = 30, 17.4%), basiliximab alone (*n* = 6, 3.5%), methotrexate-based treatment (*n* = 16, 9.3%), and ruxolitinib-based treatment (*n* = 49, 28.5%).

**Table 1:** Patient characteristics (*n* = 172)

Variable	Summary statistics
Age [years, median (range)]	30 (1–74)
Female [*n* (%)]	71 (41.3)
Underlying disease [*n* (%)]	
Hematologic malignancies	147 (85.5)
Acute leukemia	105 (61.0)
Myelodysplastic syndrome	31 (18.0)
Chronic myelomonocytic leukaemia	5 (2.9)
Others	6 (3.5)
Nonmalignant hematologic disease	25 (14.5)
Severe aplastic anemia	15 (8.7)
Others	10 (5.8)
HCT-CI score [*n* (%)]	
Low risk	146 (84.9)
Intermediate risk	23 (13.4)
High risk	3 (1.7)
Donor-recipient relationship [*n* (%)]	
Matched sibling donor	39 (22.7)
Haploidentical related donor	106 (61.6)
Unrelated donor	13 (7.6)
Umbilical cord blood	14 (8.1)
Donor-recipient sex matched [*n* (%)]	
Male to male	72 (41.9)
Male to female	54 (31.4)
Female to male	31 (18.0)
Female to female	15 (8.7)
Conditioning regimen [*n* (%)]	
Chemotherapy-based regimen	151 (87.8)
Total body irradiation-based regimen	21 (12.2)
Infused cell doses [median (range)]	
MNC (× 10^8^/kg)	9.1 (2.3–33.2)
CD34^+^ cells (× 10^6^/kg)	4.5 (0.6–17.0)
GVHD prophylaxis regimen in haploidentical donor HSCT [*n* (%)]	
ATG-based protocol	102 (59.3)
PTCy-base protocol	4 (2.3)
Engraftment [*n* (%)]	
Neutrophil	172 (100.0)
Platelet	158 (91.9)
Time from transplantation to engraftment [d, median (range)]	
Neutrophil	12 (7–43)
Platelet	14 (7–198)
Follow-up time after xenopax treatment [d, median (range)]	495 (3–1502)
Initial dose of methylprednisolone for aGVHD [*n* (%)]	
< 2 mg/(kg·d)	102 (59.3)
≥ 2 mg/(kg·d)	70 (40.7)

*HCT-CI* hematopoietic cell transplantation-comorbidity index, *MNC* mononuclear cell, *HSCT* hematopoietic stem cell transplantation, *ATG* antithymocyte globulin, *PTCy* posttransplant cyclophosphamide, *aGVHD* acute graft-versus-host disease

### Treatment response following xenopax treatment

**Fig. 1:**
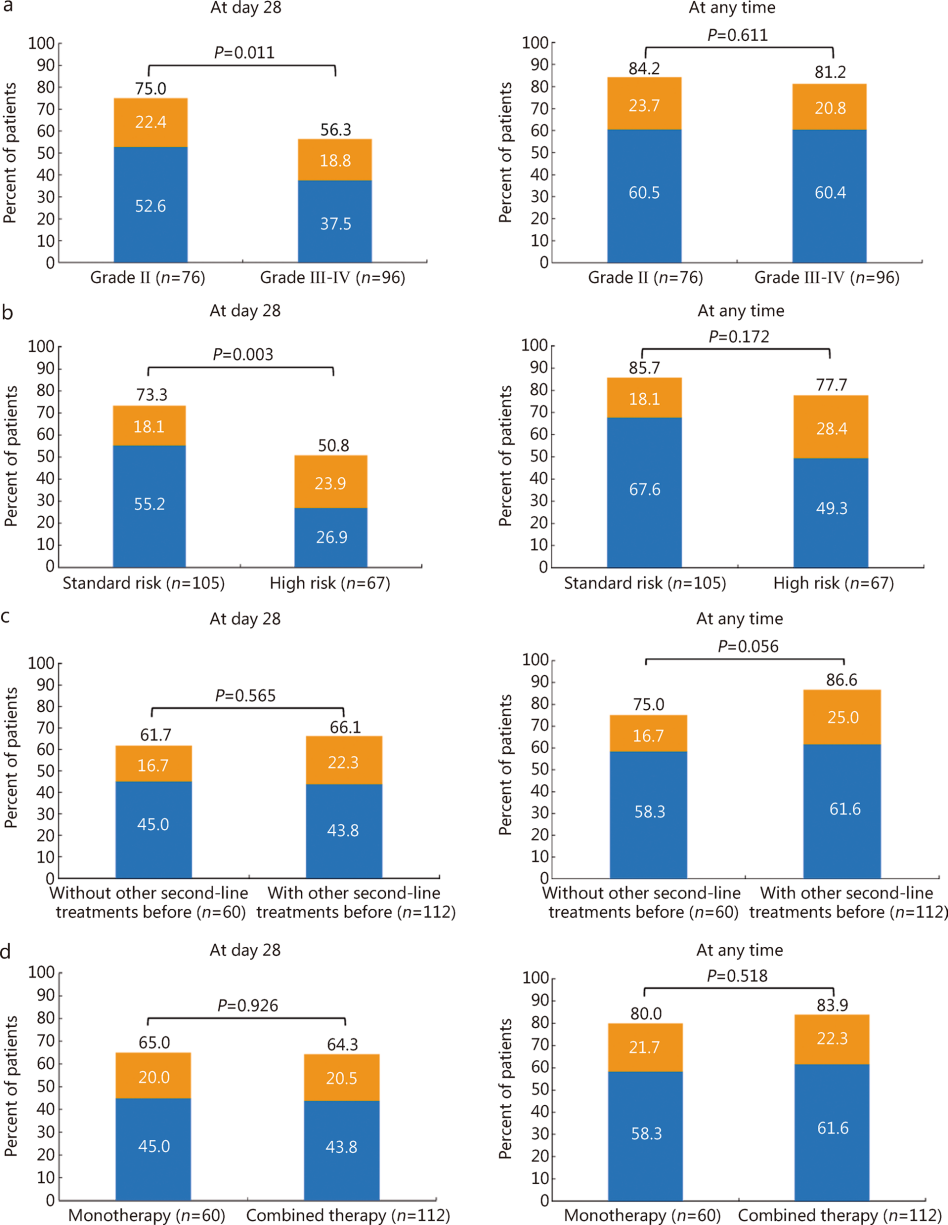
Overall response after xenopax treatment in steroid-refractory acute graft-versus-host disease (SR-aGVHD) patients. **a** At day 28 and at any time, according to severity of SR-aGVHD; **b** At day 28 and at any time, according to refined Minnesota aGVHD risk score; **c** At day 28 and at any time, according to with or without other second-line treatments prior to xenopax treatment; **d** At day 28 and at any time, according to monotherapy or combined therapy. Overall response included complete response (blue) plus partial response (orange) rates

The ORRs at days 28, 42, and 56 were 64.5% [95% confidence interval (CI) 57.4–71.7%], 69.2% (95% CI 62.3–76.1%), and 76.2% (95% CI 69.8–82.5%), respectively. The ORR, CRR, and PR rate after xenopax treatment were 82.6% (95% CI 76.9–88.2%), 60.5% (95% CI 53.2–67.8%), and 22.1% (95% CI 15.9–28.3%), respectively (Additional file 1: Table S5). Subgroup analyses of the ORR at day 28 and at any time are shown in Additional file 1: Table S6. Patients with grade III–IV aGVHD [56.3% (95% CI 46.4–66.2%) vs. 75.0% (95% CI 65.3–84.7%), *P* = 0.011] or a high-risk refined Minnesota aGVHD risk score [50.8% (95% CI 38.8–62.7%) vs. 73.3% (95% CI 64.9–81.8%), *P* = 0.003] before xenopax treatment had a lower ORR at day 28. However, the ORR at any time was comparable between patients with grade III–IV aGVHD and those with grade II aGVHD [81.2% (95% CI 73.4–89.0%) vs. 84.2% (95% CI 76.0–92.4%), *P* = 0.611], as well as between those with high- and standard-risk refined Minnesota aGVHD risk scores [77.7% (95% CI 67.6–87.6%) vs. 85.7% (95% CI 79.0–92.4%), *P* = 0.172] (Fig. 1a, b; Additional file 1: Table S6). The ORRs at day 28 and at any time between patients with or without gut involvement before xenopax treatment were 58.1% (95% CI 49.6–66.7%) vs. 83.7% (95% CI 72.8–94.6%) (*P* = 0.002) and 79.8% (95% CI 72.9–86.8%) vs. 90.7% (95% CI 82.2–99.2%) (*P* = 0.104), respectively. The CRRs at day 28 and at any time between patients with or without gut involvement before xenopax treatment were 38.0% (95% CI 29.6–46.4%) vs. 62.8% (95% CI 48.6–77.0%) (*P* = 0.005) and 57.4% (95% CI 48.9–65.9%) vs. 69.8% (95% CI 56.3–83.3%) (*P* = 0.150), respectively. We also compared the ORR at day 28 and at any time between patients receiving different conditioning regimens and those with donor-recipient relationships. These subgroup analyses revealed that, the ORRs at day 28 and at any time were 62.9% (95% CI 55.2–70.6%) vs. 76.2% (95% CI 58.0–94.4%) (*P* = 0.233) and 82.1% (95% CI 76.0–88.2%) vs. 85.7% (95% CI 70.7–100.0%) (*P* = 0.684), respectively, for patients who received chemotherapy- vs. total body irradiation-based conditioning regimens, and 59.0% (95% CI 43.6–74.4%) vs. 66.2% (95% CI 58.2–74.2%) (*P* = 0.409) and 74.4% (95% CI 60.7–88.1%) vs. 85.0% (95% CI 78.9–91.1%) (*P* = 0.125), respectively, for those undergoing matched sibling donor vs. alternative donor HSCT (Additional file 1: Table S7).

A total of 112 patients had received other second-line therapies before xenopax. The ORRs at day 28 and at any time following xenopax treatment were 66.1% (95% CI 57.3–74.9%) vs. 61.7% (95% CI 49.4–74.0%) (*P* = 0.565) and 86.6% (95% CI 80.3–92.9%) vs. 75.0% (95% CI 64.0–86.0%) (*P* = 0.056), respectively, for patients treated with and without prior second-line therapies (Fig. 1c). Specifically, for the patients who received ruxolitinib-based treatment prior to xenopax, the ORRs at day 28 and at any time were 69.4% (95% CI 56.5–82.3%) and 89.8% (95% CI 81.3–98.3%), respectively (Additional file 1: Fig. S1). For the patients who received methotrexate-based treatment before xenopax, the ORRs at day 28 and at any time were 72.3% (95% CI 50.4–94.2%) and 94.4% (95% CI 83.1–100.0%), respectively (Additional file 1: Fig. S1). In addition, the ORRs at day 28 and at any time were 60.0% (95% CI 42.5–77.5%) and 80.0% (95% CI 65.7–94.3%), respectively, for patients who received mycophenolate mofetil alone prior to xenopax; while the ORRs at day 28 and at any time were both 83.4% (95% CI 53.5–100.0%) for patients who received basiliximab alone prior to xenopax (Additional file 1: Fig. S1).

Sixty patients received xenopax monotherapy, whereas 112 patients received xenopax combined with other second-line immunosuppressants. In the combined therapy group, 47 (42.0%), 38 (33.9%), and 27 (24.1%) patients received 1, 2, and ≥ 3 types of systemic immunosuppressants, respectively, in addition to xenopax. Compared with the monotherapy group, the combined therapy group had a greater percentage of patients with grade III–IV aGVHD (64.3% vs. 40.0%, *P* = 0.002; Additional file 1: Table S8). The ORRs at day 28 and at any time following xenopax treatment were 64.3% (95% CI 55.4–73.2%) vs. 65.0% (95% CI 53.0–77.1%) (*P* = 0.926) and 83.9% (95% CI 77.1–90.7%) vs. 80.0% (95% CI 69.9–90.1%) (*P* = 0.518), respectively, for patients who received xenopax with or without other second-line therapies (Fig. 1d).

The multivariate analysis revealed that gut involvement before xenopax treatment (*P* = 0.048) and the refined Minnesota aGVHD risk score (*P* = 0.048) were related to the ORR on day 28. However, no factors were found to be associated with the ORR at any time (Table 2).

**Table 2:** Multivariate analysis for response, infection and clinical outcomes after xenopax treatment

Outcome	*HR* (95% CI)	*P*-value
Lack of response at 28 d		
Refined Minnesota aGVHD risk score before xenopax treatment		
Standard risk	1	
High risk	2.01 (1.00–4.01)	0.048
Gut involvement before xenopax treatment		
Yes	1	
No	0.39 (0.15–0.99)	0.048
Infection		
Refined Minnesota aGVHD risk score before xenopax treatment		
Standard risk	1	
High risk	3.79 (1.97–7.27)	< 0.001
Treatment failure as defined by OS		
Donor type		
Matched sibling donor	1	
Others	0.54 (0.30–0.94)	0.030
HCT-CI score		
0 score	1	
≥ 1 score	2.52 (1.39–4.56)	0.020
Treatment failure as defined by DFS		
Age		
< 18 years	1	
≥ 18 years	3.08 (1.69–5.62)	< 0.001
Donor type		
Matched sibling donor	1	
Others	0.54 (0.333–0.88)	0.014
Relapse		
Age		
< 18 years	1	
≥ 18 years	4.27 (1.27–14.40)	0.019
Donor-recipient sex matched		
Female to male	1	
Others	0.33 (0.16–0.69)	0.003
Severity of aGVHD at diagnosis		
Grade II	1	
Grade III–IV	0.22 (0.08–0.59)	0.002
NRM		
HCT-CI score		
0 score	1	
≥ 1 score	3.15 (1.68–5.91)	< 0.001
Refined Minnesota aGVHD risk score before xenopax treatment		
Standard risk	1	
High risk	2.07 (1.14–3.78)	0.017

Multivariate analysis for relapse only enrolled the patients with hematologic malignancies. No factors were associated with lack of response at any time in multivariate analysis. *OS* overall survival, *HCT-CI* hematopoietic cell transplantation-comorbidity index, *DFS* disease-free survival, *aGVHD* acute graft-versus-host disease, *HR* hazard ratio, *CI* confidence interval, *NRM* non-relapse mortality

### Toxicities and infections following xenopax treatment

No allergic or infusion reactions were reported during the administration of xenopax. The details of the infections that occurred after xenopax treatment are summarized in Table 3. The percentages of new-onset viral, bacterial, and fungal infections were 23.3%, 16.3%, and 5.8%, respectively. The percentages of patients with any infection (≥ 1 type) and those with multiple infections (≥ 2 types) were 37.8% and 12.8%, respectively. Infection rates, particularly for viral and bacterial infections, were higher among patients receiving the combined therapy than among those receiving monotherapy. Additionally, viral infections (30.2% vs. 14.5%, *P* = 0.015) and any infection (≥ 1 type; 47.9% vs. 25.0%, *P* = 0.002) were more common in patients with grade III–IV aGVHD than in patients with grade II aGVHD. Infection rates, including viral (27.7% vs. 15.0%, *P* = 0.061), bacterial (18.8% vs. 11.7%, *P* = 0.230), fungal (6.3% vs. 5.0%, *P* = 0.738), any infection (38.4% vs. 36.7%, *P* = 0.824), and multiple infection (15.2% vs. 8.3%, *P* = 0.200), were all comparable between patients who received xenopax with and without prior second-line therapies (Additional file 1: Table S9). The multivariate analysis showed that a high-risk Minnesota aGVHD risk score before xenopax treatment was associated with a greater risk of infection (*P* < 0.001; Table 2).

**Table 3:** New onset infections after xenopax treatment

Types of infection	*n* (%)
Viral infection	40 (23.3)
Cytomegalovirus infection	34 (19.8)
Cytomegalovirus DNAemia	32 (18.6)
Cytomegalovirus disease	2 (1.2)
Epstein-Barr virus DNAemia	12 (7.0)
Other viremias	4 (2.3)
Bacterial infection	28 (16.3)
Sepsis	10 (5.8)
Pneumonia	12 (7.0)
Central nervous system	1 (0.6)
Urinary tract	2 (1.2)
Other sites	5 (2.9)
Fungal infection	10 (5.8)
Pneumonia	8 (4.7)
Other sites	2 (1.2)
Any infection (≥ 1 type)	65 (37.8)
Multiple infection (≥ 2 types)	22 (12.8)

*DNA* deoxyribonucleic acid

### Steroid use

**Fig. 2:**
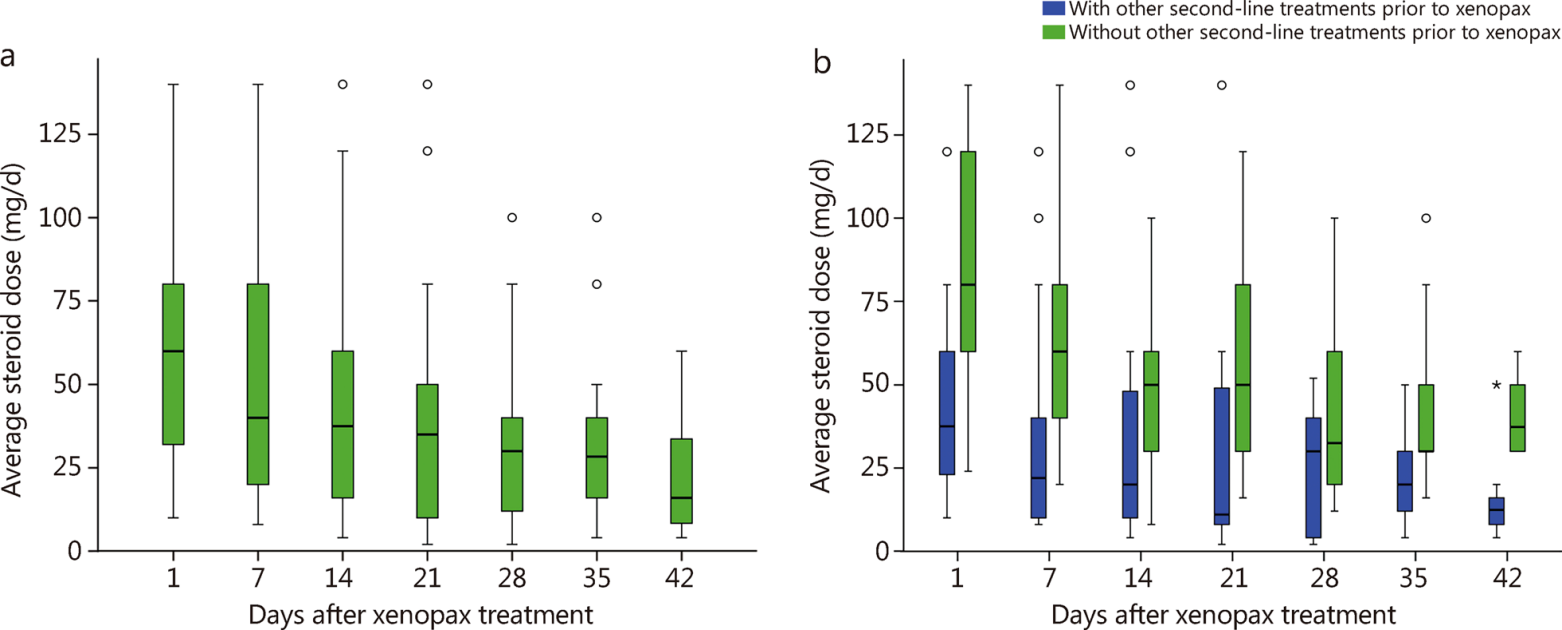
Steroid dose over time. The steroid dose at days 1, 7, 14, 21, 28, 35 and 42 is displayed for all patients (**a**) and those who with/without other second-line treatment prior to xenopax (**b**). Data showed median (horizontal line), 75th and 25th quartiles (upper and lower boundaries, respectively), and minimum (lower error bar)/maximum (upper error bar)

The median initial steroid dose (methylprednisolone dose, mg) at the beginning of xenopax treatment was 60.0 mg/d. The steroid dose decreased gradually over time, and the median steroid doses at 7, 14, 21, and 28 d after xenopax were 40.0, 37.5, 35.0, and 30.0 mg, respectively, which suggested that 58.1% (18/31) of patients receiving xenopax had a 50% or greater reduction in the baseline steroid dose by day 28 (Fig. 2a, b).

### cGVHD after xenopax treatment

No flare-ups of aGVHD were observed after discontinuation of xenopax. Throughout the follow-up period, 30 patients developed cGVHD (Additional file 1: Table S10). The median time from the start of xenopax treatment to the onset of cGVHD was 120 d (range, 7–555 d). The cumulative incidences of overall and moderate-to-severe cGVHD at 2 years after xenopax treatment were 12.9% (95% CI 7.8–18.0%) and 5.2% (95% CI 4.4–7.4%), respectively.

### Other clinical outcomes following xenopax treatment

The 2-year probabilities of relapse, NRM, DFS, and OS following xenopax treatment were 19.0% (95% CI 12.8−25.2%), 24.2% (95% CI 18.0−30.9%), 57.0% (95% CI 49.9 −65.0%), and 68.0% (95% CI 61.4 −75.4%), respectively (Additional file 1: Fig. S2), and the clinical outcomes in different subgroups are shown in Additional file 1: Table S11, while the causes of mortality are presented in Additional file 1: Table S12. We observed that the conditioning regimen (chemotherapy-based regimen vs. total body irradiation-based regimen) had no effect on OS or NRM, but the alternative donor group had a higher 2-year OS rate (72.2% vs. 53.8%, *P* = 0.027) and a marginally lower NRM rate (23.1% vs. 38.1%, *P* = 0.069) compared with the matched sibling donor group (Additional file 1: Table S7). In the multivariate analysis, a high-risk Minnesota aGVHD risk score was related to a higher risk of NRM, whereas severe aGVHD (grade III–IV) was not associated with a higher risk of NRM or worse survival outcomes (Table 2; Additional file 1: Table S11).

### Comparison between xenopax and other second-line treatments

#### Efficacy

We compared xenopax with other second-line treatments for SR-aGVHD (Additional file 1: Methods) in independent historical cohorts, and most of the characteristics were comparable between the groups (Additional file 1: Tables S13-S17). The ORR at day 28 [64.5% (95% CI 57.3–71.7%) vs. 70.4% (95% CI 67.6–73.2%),* P* = 0.125] and at any time [82.6% (95% CI 76.9–88.3%) vs. 77.5% (95% CI 74.9–80.1%),* P* = 0.137] were both comparable between the xenopax and BATs groups. In the subgroup analysis, the xenopax group presented a significantly better ORR at day 28 and at any time than that of the MSCs group. In addition, the xenopax group showed a better ORR at any time than that of the ruxolitinib (*P* = 0.042) group or the MSCs plus basiliximab group (*P* = 0.014) (Additional file 1: Fig. S3a-e). The ORR between xenopax in the present study and ruxolitinib in the REACH1 study (at day 28: 64.5% vs. 55.0%; at any time: 82.6% vs. 73.2%) is shown in Additional file 1: Table S18. In the multivariate analysis, grade III–IV aGVHD was associated with a worse ORR at day 28 (*P* < 0.001), and no factor was associated with ORR at any time (Additional file 1: Table S19).

In the sensitivity analysis, xenopax showed a trend toward a better ORR at any time compared with BATs [82.6% (95% CI 76.9–88.3%) vs. 76.3% (95% CI 74.3–79.2%), *P* = 0.075], and the ORR at day 28 [64.5% (95% CI 57.3–71.7%) vs. 67.9% (95% CI 64.7–71.1%), *P* = 0.399] was similar between the groups in the propensity score matching (PSM) analysis.

#### Infections

The rate of at least one infection event was lower in the xenopax group than in the BATs group (37.8% vs. 60.8%, *P* < 0.001). In the subgroup analysis, the rate of at least one infection event in the xenopax group was similar to those in the MSCs group (35.7%, *P* = 0.225) or ruxolitinib group (46.7%, *P* = 0.497), which was lower than patients in the basiliximab group (60.7%, *P* < 0.001) or MSCs plus basiliximab group (80.0%, *P* < 0.001). The rate of at least one infection event was 80.3% in the REACH1 study. In the multivariate analysis, receiving BATs and grade III–IV aGVHD before second-line treatment was associated with a higher risk of infection (Additional file 1: Table S19).

In the sensitivity analysis, the rate of at least one infection event was lower in the xenopax group than in the BATs group in the PSM analysis (37.8% vs. 63.1%, *P* < 0.001).

#### OS and NRM

The 2-year OS and NRM rates after treatment were 68.0% (95% CI 61.4−75.4%) vs. 65.3% (95% CI 62.4−68.2%) (*P* = 0.795), and 24.2% (95% CI 17.5 −30.9%) vs. 28.0% (95% CI 25.1− 30.9%) (*P* = 0.784), respectively, in the xenopax group and BATs group (Additional file 1: Fig. S4). In the subgroup analysis, the 2-year OS and NRM rates after treatment were comparable between groups receiving xenopax and other second-line treatments (Additional file 1: Fig. S5). The 1-year OS and NRM rates were 42.6% (95% CI 30.0−54.6%) and 52.9% (95% CI 39.6−64.5%), respectively, in the REACH1 study (Additional file 1: Table S18). In the multivariate analysis, older patients, a higher HCT-CI score, and grade III–IV aGVHD were associated with a higher risk of lower 2-year OS and higher 2-year NRM rates (Additional file 1: Table S19).

In the sensitivity analysis, the 2-year OS and NRM rates were both comparable between the xenopax and BATs groups in the PSM analysis (Additional file 1: Fig. S6).

#### Cost-effectiveness analysis

When incorporating ORR at any time, the median effective number of doses, and unit drug costs of basiliximab, MSCs, ruxolitinib, and xenopax, the incremental cost per additional responder for SR-aGVHD patients was 40,539, 42,031, 3136, and 31,780 RMB for these drugs, respectively (Additional file 1: Table S20).

## Discussion

In this large-scale study of 172 SR-aGVHD patients receiving xenopax, we found an ORR of 64.5% on day 28 (Additional file 1: Table S5), with 2-year OS and NRM rates of 68.0% and 24.2% (Additional file 1: Fig. S2), respectively. We also observed that xenopax showed some superior features over other second-line treatments. To our knowledge, this is one of the largest studies to validate the efficacy of a humanized IL-2R antagonist for the treatment of SR-aGVHD under real-world conditions.

We observed that the ORR at any time was 81.2% and 77.7% (Fig. 1), respectively, for patients who had grade III–IV aGVHD (*n* = 96, 55.8%) and high-risk Minnesota aGVHD (*n* = 67, 39.0%). For other systemic second-line therapies, the reported ORR ranged from 33% to 55% [6, 31] and 60.6% to 67.4% [13], respectively, for patients with grade III–IV aGVHD and those in the high-risk Minnesota aGVHD group. Importantly, we found that severe aGVHD and high-risk Minnesota aGVHD, which were both related to a decreased ORR and shorter survival of SR-aGVHD patients treated with basiliximab [13], did not negatively impact the ORR at any time or survival following xenopax treatment. These findings further support the efficacy of xenopax in treating severe SR-aGVHD.

We observed that xenopax has some advantages over basiliximab. For example, as a humanized antibody, xenopax may induce a weaker immune response. In addition, the elimination half-life of xenopax is nearly twice as long as that of basiliximab (13.3 d vs. 7.2 d) [34, 35], suggesting that xenopax could suppress T cells more persistently. We also observed that xenopax had a lower risk of infection compared with basiliximab. Finally, as we observed that xenopax showed a better cost-effectiveness compared with basiliximab, it may lay a foundation for reducing the economic burden of Chinese patients with SR-aGVHD.

Based on the findings from the REACH studies [27, 31] and several meta-analyses [36–38], ruxolitinib has been recommended for the treatment of SR-aGVHD [39–41]. Because neither the REACH1 [31] nor REACH2 [27] study enrolled Chinese patients, the efficacy and safety of ruxolitinib had not been fully assessed in China. Thus, no standard second-line treatments were recommended by the Chinese consensus [24, 40], although ruxolitinib has been approved by NMPA for SR-aGVHD. Considering the high ORR of xenopax treatment, designing an RCT to further compare the clinical outcomes between xenopax and ruxolitinib in China is worthwhile. In addition, the 28-day and sustained 56-day CRR after ruxolitinib were only 34.4% and 26.6%, respectively, in the REACH2 study [27]. The ORRs at day 28 and at any time after xenopax treatment were 69.4% and 89.8%, respectively, for those who showed no response to ruxolitinib-based treatment in the present study (Additional file 1: Fig. S1), which suggested that xenopax could also be used as a salvage therapy for patients who fail to respond to ruxolitinib treatment.

Methotrexate was another important treatment for patients with SR-aGVHD [40]. However, approximately 40% of these patients showed a response to methotrexate treatment [42]. Recently, Zhang et al. [43] reported that compared with a single-dose xenopax plus methotrexate regimen, a double-dose xenopax regimen without methotrexate reduces the cumulative incidence of total and grade III–IV aGVHD by day 100, as well as the cumulative incidence of total and moderate/severe cGVHD at 1 year. In the present study, the ORR at day 28 and at any time after xenopax was 72.3% and 94.4%, respectively, for those who showed no response to methotrexate-based second-line treatments (Additional file 1: Fig. S1), which further suggested that xenopax could be used as the salvage therapy for these patients. In addition, some novel agents (e.g., glucagon-like peptide-2 inhibitor, A1-antitrypsin, and receptor-interacting protein kinase inhibitor) have been tried to treat SR-aGVHD, which can be used with xenopax and help further improve the outcomes of SR-aGVHD patients [41].

In the present study, the ORR at day 28 and at any time was 59.0% and 74.4%, respectively, for those receiving matched sibling donor after xenopax treatment (Additional file 1: Table S6), which was similar to the results of patients receiving basiliximab treatment (ORR at day 28: 54.0%; ORR at any time: 63.5%) [12]. In addition, we observed that the ORR at day 28 after xenopax treatment was 59.0%, 66.0%, and 61.5%, respectively, for matched sibling donor, haploidentical related donor, and unrelated donor groups in the present study (Additional file 1: Table S6). Similarly, the ORR at day 28 after basiliximab treatment was 61.0%, 73.3%, and 62.3%, respectively, for matched sibling donor, haploidentical related donor, and unrelated donor groups [17]. Thus, we suggested that donor type did not impact the clinical outcomes after IL-2R antagonist treatment in SR-aGVHD patients.

Severe aGVHD was the most important risk factor for cGVHD [44]. Lastovytska et al. [45] reported that ruxolitinib plus extracorporeal photopheresis had a higher ORR, which led to less cGVHD at 1 year compared to ruxolitinib alone in SR-aGVHD patients. This could also partially contribute to the lower incidence of total (12.9%) and moderate to severe cGVHD (5.2%) in the present study.

Infection events are common in SR-aGVHD patients, and 39.1% to 80.3% of patients will experience at least 1 infection event following second-line treatment [17, 31, 46–48]. In this study, only 37.8% of patients (65/172) developed at least one infection following xenopax treatment (Table 3), which was lower than patients receiving BATs in the historical cohort (Additional file 1: Table S19). Particularly, half of the patients (34/65) experienced cytomegalovirus (CMV) infections, and most patients did not routinely receive letermovir for CMV prophylaxis. Thus, we anticipate that the CMV activation rate would decrease in the era of letermovir, which may further decrease the overall infection rate after xenopax treatment. On the other hand, the median number of injections of xenopax administered in this study was only 3, and the infection risk increased significantly after the fifth or sixth injection of the IL-2R antagonist. Rapidly controlling GVHD via xenopax treatment could also contribute to a lower infection rate after treatment.

Patients with severe SR-aGVHD often receive multiple therapies to increase the response rate [47, 49, 50], which may increase the likelihood of infection [48]. In this study, we observed a higher percentage of patients with grade III–IV aGVHD in the combined therapy group than in the monotherapy group. Given that the ORR was similar between the groups, combining xenopax with other second-line therapies may help mitigate the adverse effects of grade III–IV aGVHD on the therapeutic response and survival. However, the infection rate was higher in patients receiving combined therapies than in those receiving xenopax monotherapy [17]. Thus, although the infection risk of xenopax treatment was relatively low, attention should still be given to patients receiving xenopax combined with other immunosuppressants. On the other hand, several studies have shown that combining an IL-2R antagonist (e.g., basiliximab) with specific second-line treatments, such as MSCs [46, 51], methotrexate, and vedolizumab [52], can achieve a higher ORR without significantly increasing infections, which could be further explored in patients receiving xenopax treatment.

We did not identify the optimal protocols for xenopax using machine learning because of the relatively small sample in the present study. According to the study of basiliximab [17], the increase in infection overcomes the benefit of ORR after the third and the fifth dose of basiliximab, respectively, in patients with grade II and grade III–IV SR-aGVHD. So, we suggested that if grade II and grade III–IV patients showed no response after the third and the fifth dose of xenopax, respectively, the treatment should be stopped. If the patients achieved PR during the treatment, xenopax can be continued when the aGVHD is less than grade II. These could be further confirmed in a prospective large-scale study.

Our study has several limitations. First, the median age of the patients in this study was only 30 years, which could restrict the generalizability of our findings to older populations. Nevertheless, the ORRs at day 28 and at any time were as high as 60.5% and 76.3%, respectively, in patients older than 50 years, suggesting that older patients could also benefit from xenopax treatment. Second, over 60% of the patients underwent HSCT with haploidentical related donors, with nearly all haploidentical related donor HSCT recipients receiving ATG for GVHD prevention, whereas only 4 patients underwent treatment with post-transplantation cyclophosphamide (PTCy). Therefore, the safety and efficacy of xenopax in patients receiving PTCy for GVHD prophylaxis should be further explored in larger studies. Third, most of the centers did not routinely detect the immune cell subsets or cytokine levels before and after xenopax treatment in this real-world study, which should be further identified in our prospective study in the future. Finally, although we compared the efficacy, infection, NRM, OS, and cost-effectiveness between xenopax and BATs in independent historical cohorts, however, the BATs cohort was heterogeneous. For example, the treatments, as well as the inclusion and exclusion criteria were different, which could not be matched completely. Thus, the safety and efficacy of xenopax should be further confirmed by prospective, multicenter RCTs in the future.

## Conclusions

Thus, the findings of our real-world analysis support the safety and efficacy of xenopax for the treatment of SR-aGVHD. In future prospective RCTs, further comparisons of the clinical outcomes between xenopax and other systemic second-line therapies could be performed.

## Supplementary Information

**Additional file 1. Methods. Table S1** Comparison between xenopax and basiliximab. **Table S2** Transplant regimens. **Table S3** Second-line treatments of other studies. **Table S4** Characteristics of aGVHD patients (*n* = 172). **Table S5** Overall response at different time after xenopax treatment between different groups. **Table S6** Subgroup analysis for overall response rate (ORR) at day 28 and at any time after xenopax treatment. **Table S7** Overall response rate, OS and NRM in different subgroups after xenopax treatment. **Table S8** Patient characteristics between monotherapy and combined therapy groups. **Table S9** New onset infections after xenopax treatment in subgroup analysis [n (%)]. **Table S10** Characteristics of cGVHD (*n* = 30). **Table S11** Clinical outcomes at 2 years after xenopax treatment in subgroup analysis [cumulative incidence, % (95% CI)]. **Table S12** Causes of death. **Table S13** Patient characteristics between best available treatments (BATs) and xenopax groups. **Table S14** Patient characteristics between basiliximab and xenopax groups. **Table S15** Patient characteristics between mesenchymal stromal cells (MSCs) and xenopax groups. **Table S16** Patient characteristics between mesenchymal stromal cells (MSCs) plus basiliximab and xenopax groups. **Table S17** Patient characteristics between ruxolitinib and xenopax groups. **Table S18** The comparison of enrollment time, second-line treatment for SR-aGVHD, infection rate, OS and NRM between ruxolitinb (REACH1) and xenopax. **Table S19** Univariate and multivariate analysis for response and clinical outcomes in total cohort with steroid-refractory acute graft-versus-host disease after second-line treatments. **Table S20** The cost of different drugs during treatment of acute GVHD. **Fig. S1** Overall response of patients receiving other second-line treatments before xenopax at day 28 and at any time. **Fig. S2** Clinical outcomes at 2 years after xenopax treatment. **Fig. S3** The overall response at day 28 and at any time of (a) xenopax vs. best available treatments (BATs), (b) xenopax vs. basiliximab, (c) xenopax vs. mesenchymal stromal cells (MSCs), (d) xenopax vs. ruxolitinib, and (e) xenopax vs. MSCs plus basiliximab. **Fig. S4** Clinical outcomes at 2 years after xenopax and best available treatments (BATs) treatment. **Fig. S5** Clinical outcomes at 2 years after xenopax and best available treatments (BATs). **Fig. S6** Clinical outcomes at 2 years between xenopax and best available treatments (BATs) groups in the propensity score matching (PSM) analysis.

## Data Availability

The dataset supporting the conclusions of this article is available in the clinical data repository of each participating hospital. Individual participant data were not shared. For the original data, please contact moxiaodong@pkuph.edu.cn.
