# Supplementary material for: Xenopax for the treatment of steroid-refractory acute graft-versus-host disease: the RELAX study
Source: Mil Med Res. 2025 Sep 29;12:63. doi: 10.1186/s40779-025-00640-0 (PMC12477784; doi:10.1186/s40779-025-00640-0)
Supplement: Supplementary file 1 — Additional file 1. Methods. Table S1 Comparison between xenopax and basiliximab. Table S2 Transplant regimens. Table S3 Second-line treatments of other studies. Table S4 Characteristics of aGVHD patients (n = 172). Table S5 Overall response at different time after xenopax treatment between different groups. Table S6 Subgroup analysis for overall response rate (ORR) at day 28 and at any time after xenopax treatment. Table S7 Overall response rate, OS and NRM in different subgroups after xenopax treatment. Table S8 Patient characteristics between monotherapy and combined therapy groups. Table S9 New onset infections after xenopax treatment in subgroup analysis [n (%)]. Table S10 Characteristics of cGVHD (n = 30). Table S11 Clinical outcomes at 2 years after xenopax treatment in subgroup analysis [cumulative incidence, % (95% CI)]. Table S12 Causes of death. Table S13 Patient characteristics between best available treatments (BATs) and xenopax groups. Table S14 Patient characteristics between basiliximab and xenopax groups. Table S15 Patient characteristics between mesenchymal stromal cells (MSCs) and xenopax groups. Table S16 Patient characteristics between mesenchymal stromal cells (MSCs) plus basiliximab and xenopax groups. Table S17 Patient characteristics between ruxolitinib and xenopax groups. Table S18 The comparison of enrollment time, second-line treatment for SR-aGVHD, infection rate, OS and NRM between ruxolitinb (REACH1) and xenopax. Table S19 Univariate and multivariate analysis for response and clinical outcomes in total cohort with steroid-refractory acute graft-versus-host disease after second-line treatments. Table S20 The cost of different drugs during treatment of acute GVHD. Fig. S1 Overall response of patients receiving other second-line treatments before xenopax at day 28 and at any time. Fig. S2 Clinical outcomes at 2 years after xenopax treatment. Fig. S3 The overall response at day 28 and at any time of (a) xenopax vs. best available treatments [file 40779_2025_640_MOESM1_ESM.pdf]

Methods

Data collection

The necessary data of each participating hospital included patient demographics, diagnoses, transplantation protocols [e.g., human leukocyte antigen (HLA) disparity, donor type, and graft-versus-host disease (GVHD) prophylaxis], details of acute GVHD (aGVHD; affected organs, severity, and refined Minnesota aGVHD risk score), detail of xenopax treatment (clinical outcomes and toxicities), chronic GVHD (cGVHD), mortality, and survival.

Protocol of other second-line treatments before, or combined with xenopax

Mycophenolate mofetil: 0.5, 1.0, and 1.5 g/d for patients weighing less than 40 kg, more than 40 kg but less than 70 kg, and more than 70 kg [1].

Ruxolitinib was given at a dose of 5 mg orally, twice daily (BID), for patients weighing ≤ 50 kg, and 10 mg BID for patients weighing > 50 kg. For children weighing ≤ 25 kg, it was recommended at 2.5 mg BID, and for children weighing > 25 kg, it was 5 mg BID [2].

Methotrexate: 5 mg/m^2 on days 1, 3, or 8 and then weekly [3].

Mesenchymal stromal cells (MSCs) were given intravenously at a dose of 1×10^6 cells/kg once weekly for 4 consecutive weeks as a cycle [4].

Response assessments for aGVHD treatments

Complete response (CR) is defined as a score of 0 for the aGVHD grading in all evaluable organs that indicates complete resolution of all signs and symptoms of aGvHD in all evaluable organs without administration of additional systemic therapy for any earlier progression, mixed response, or non-response of aGVHD. Partial response (PR) is defined as improvement of 1 stage in 1 or more organs involved with aGVHD signs or symptoms without progression in other organs or sites without administration of additional systemic therapy for an earlier progression, mixed response, or non-response of aGVHD.

Overall response rate (ORR) includes CR plus PR rates. Lack of response is defined as no response, mixed response, or progression. No response is defined as the absence of improvement in any organ involved by aGVHD, without worsening in any involved organ. Progression is defined as worsening in 1 or more organs by 1 or more stages without improvement in any involved organ.

Particularly, patients requiring additional systemic therapy for aGVHD will be classified as a lack of response.

Endpoints and definition

Overall survival (OS): time from xenopax treatment to death from any cause; disease-free survival (DFS): survival period with continuous complete remission after xenopax treatment; non-relapse mortality (NRM): time from xenopax treatment to death from any cause without recurrence of underlying disease; relapse: time from xenopax treatment to relapse.

Sample size calculation of this study

Power Analysis and Sample Size software (PASS 2023) was used to evaluate the statistical power or sample size of our study. This study began on January 1, 2020. At the time this study was initiated, ruxolitinib had not yet been approved by the National Medical Products Administration of China for the treatment of aGVHD (It was approved after April 2023 in China).

Therefore, we estimated the sample size mainly based on the best available treatments (BATs) group of the REACH2 study (such as antithymocyte globulin, MSCs, methotrexate, mycophenolate mofetil, sirolimus, and so on) [5], which also reflected the standard of care for aGVHD in China when this study began. Notably, the BATs group in REACH2 did not include IL-2 receptor antagonists, further supporting the rationale for using this group as a reference for sample size estimation.

The present study was planned to detect a 28-day ORR of 49% (i.e., a 10% increase) in patients receiving xenopax treatment, from the reference rate of the REACH2 study of 39%, controlling for type I and II error rates at 5% and 20%, respectively. Considering an expulsion rate of 15%, a total of 172 patients were planned to be enrolled.

Multivariate analysis

Hazard ratios (*HR*s) for ORR, mortality, and survival were estimated in a multivariate analysis using Cox proportional hazards regression. Independent variables with $P > 0.1$ were sequentially excluded from the model, and $P < 0.05$ was considered to be statistically significant. The following variables were included: gender, patient age (< 18 years vs. ≥ 18 years), underlying disease (hematologic malignancies vs. nonmalignant hematologic disease), hematopoietic cell transplantation-specific comorbidity index (intermediate-high risk vs. low-risk), donor-recipients gender matched (female to male vs. others), donor type (matched sibling donor vs. alternative donor), donor-recipient relation (maternal donors vs. other donors), donor-recipient blood group matched (mismatched vs. matched), mononuclear and $CD34^+$ cell counts (using the mean value as the cut-off point), initial dose of steroid [≥ 2 mg/(kg·d) vs. methylprednisolone < 2 mg/(kg·d)], steroid refractory type (steroid resistant vs. steroid dependent), severity of aGVHD at diagnosis and at the beginning of second-line treatment (≥ grade III vs. < grade III), refined Minnesota aGVHD risk score at diagnosis and at the beginning of second-line treatment (high-

risk vs. standard-risk), and protocol of second-line treatment (combined therapy vs. monotherapy in xenopax cohort; xenopax vs. BATs in total cohort). Independent variables with $P > 0.1$ were sequentially excluded from the model, and $P < 0.05$ was considered to be statistically significant.

List of investigators

No.	Investigator name	Center name	Center location
1	Xiao-Dong Mo, Le-Qing Cao, Wen-Xuan Huo	Peking University People's Hospital, Peking University Institute of Hematology, National Clinical Research Center for Hematologic Disease, Beijing Key Laboratory of Cell and Gene Therapy for Hematologic Malignancies	Beijing, China
2	Er-Lie Jiang	State Key Laboratory of Experimental Hematology, National Clinical Research Center for Blood Diseases, Haihe Laboratory of Cell Ecosystem, Institute of Hematology & Blood Diseases Hospital, Chinese Academy of Medical Sciences & Peking Union Medical College	Tianjin, China
3	Yue-Wen Fu	Henan Cancer Hospital, Affiliated Cancer Hospital of Zhengzhou University	Zhengzhou, Henan, China
4	Xiao-Jun Xu	Children's Hospital, Zhejiang University School of Medicine and National Clinical Research Center for Child Health	Hangzhou, Zhejiang, China
5	Ping-Chong Lei	Henan Provincial People's Hospital	Zhengzhou, Henan, China
6	Ming-Feng Zhao	Tianjin First Central Hospital	Tianjin, China
7	Zhi Chen	Department of Hematology, Wuhan Children's Hospital (Wuhan Maternal and Child Healthcare Hospital), Tongji Medical College, Huazhong University of Science & Technology	Wuhan, Hubei, China
8	Shu-Xia Guo	People's Hospital of Zhengzhou	Zhengzhou, Henan, China
9	Xiao-Bing Huang	Sichuan Provincial People's Hospital, Affiliated Hospital of University of Electronic Science and Technology of China	Chengdu, Sichuan, China
10	Yan-Ming Zhang	Huai'an Second People's Hospital	Huai'an, Jiangsu, China
11	Xian-Jing Wang	The Third People's Hospital of Zhengzhou	Zhengzhou, Henan, China
12	Guan-Chen Bai	Department of Hematology, the Affiliated Tai'an City Central Hospital of Qingdao University	Tai'an, Shandong, China
13	Feng-Bo Jin	Anhui Public Health Clinical Center, the First Affiliated Hospital of Anhui Medical University	Hefei, Anhui, China
14	Qing-Sheng Li	The First Affiliated Hospital of Anhui Medical University	Hefei, Anhui, China

No.	Investigator name	Center name	Center location
15	Ming-Yang Deng	Department of Hematology, the Second Xiangya Hospital, Central South University	Changsha, Hunan, China
16	Hao Zhang	Affiliated Hospital of Jining Medical University	Jining, Shandong, China
17	Xin-Feng Wang	Affiliated Hospital of Nantong University	Nantong, Jiangsu, China

Table S1 Comparison between xenopax and basiliximab

Parameters	Xenopax	Basiliximab
Molecular weight (kD)	144	144
Proportion of human sequence (%)	90	75 [6]
IgG type	IgG1	IgG1
Half life (d)	13.3	7.2
C_{max} (μg/ml)	14.5*	7.1**
AUC (μg·d/ml)	254	24.7 [7]

*The C_{max} after the first dose of xenopax (1 mg/kg); **The C_{max} following intravenous infusion of 20 mg basiliximab over 30 min. *IgG* immunoglobulin G, C_{max} maximum concentration, *AUC* area under the serum concentration-time curve

Table S2 Transplant regimens

Regimen category	Regimen details
Conditioning regimen*	
Hematologic malignancies	
Standard MAC	Arac1Bu3Cy2MeCCNU1 ± Flu5/TBI1Cy2MeCCNU1
RIC/RTC	Flu5Cy2/Flu5Mel2/Arac1Bu3Flu5MeCCNU1 ± Cy2/Bu3Flu3TT1
ITC	Dec2Arac1Bu3Cy2MeCCNU1 ± Flu5/Ida3Bu3Cy2/Dec5Arac3Bu3Flu3Cy2/Cla5Arac5Bu4MeCCNU1/Cla5Bu4Cy2
Nonmalignant hematologic disease	Bu2Cy4 ± Flu5/Cy4Flu5/Cy2Flu4
Stem cell mobilization regimen of donors	G-CSF 5 μg/(kg·d) continuous subcutaneous injection for 5 – 6 d, and collecting peripheral blood stem cells and/or bone marrow stem cells
GVHD prophylaxis	All subjects received calcineurin inhibitor (ciclosporin or tacrolimus), mycophenolate mofetil, and short-term methotrexate. ATG, 1.5 to 2.5 mg/kg per day on days −5 to −2 for patients who received haploidentical donor HSCT or unrelated donor HSCT; ATG, 1.5 mg/kg per day on days −5 to −3 for patients ≥ 40 years who received matched sibling donor HSCT<br>Patients who received haploidentical HSCT could also receive PTCy on days +3 and +4 at a dose of 50 mg/kg for GVHD prophylaxis

*In the conditioning regimen, arabic numerals presented the days of drug administration. *MAC* myeloablative conditioning regimens, *Arac* cytarabine, *Bu* busulfan, *Cy* cyclophosphamide, *MeCCNU* semustine, *Flu* fludarabine, *TBI* total body irradiation, *RIC* reduced intensity conditioning regimens, *RTC* reduced toxicity conditioning regimen, *Mel* melphan, *TT* thiotepa, *ITC* intensified conditioning regimens, *Dec* decitabine, *Ida* idarubicin, *Cla* cladribine, *G-CSF* granulocyte colony-stimulating factor, *GVHD* graft-versus-host disease, *ATG* thymoglobulin, *HSCT* hematopoietic stem cell transplantation, *PTCy* posttransplant cyclophosphamide

Table S3 Second-line treatments of other studies

References	Second-line therapeutic regimen
Basiliximab [8]	Basiliximab was administered at a dose of 20 mg in adults or children weighing 35 kg or more, and at 10 mg in children weighing less than 35 kg, on days 1, 3 or 4, and 8, and repeated weekly thereafter until the aGVHD was less than grade II
Ruxolitinib [9]	Patients received a starting oral dose of ruxolitinib at 5 mg twice daily, with an option to increase to 10 mg twice daily after 3 d in the absence of cytopenias (≥ 50% decrease in platelet counts and/or absolute neutrophil count relative to day 1)
MSCs [10]	MSCs were given intravenously at a dose of (1.0 – 2.0) × 10^6/kg, once a week for 2 – 6 weeks
MSCs plus basiliximab [11]	MSCs were given an intravenous infusion of MSCs of 1.0 × 10^6 cells/kg twice a week for 4 weeks Basiliximab was administered at a dose of 20 mg on days 1, 3, or 4, and 8, and repeated weekly

aGVHD acute graft-versus-host disease, *MSCs* mesenchymal stromal cells

Table S4 Characteristics of aGVHD patients (*n* = 172)

Characteristics of aGVHD	Summary statistics
Time from diagnosis of aGVHD to xenopax treatment [d, median (range)]	7 (3 – 54)
Severity of skin aGVHD at diagnosis [*n* (%)]	
Stage 0	88 (51.2)
Stage 1	20 (11.6)
Stage 2	38 (22.1)
Stage 3	24 (14.0)
Stage 4	2 (1.2)
Severity of gut aGVHD at diagnosis [*n* (%)]	
Stage 0	52 (30.3)
Stage 1	17 (9.9)
Stage 2	51 (29.7)
Stage 3	28 (16.3)
Stage 4	24 (14.0)
Severity of liver aGVHD at diagnosis [*n* (%)]	
Stage 0	116 (67.4)
Stage 1	7 (4.1)
Stage 2	32 (18.6)
Stage 3	15 (8.7)
Stage 4	2 (1.2)
Overall severity of aGVHD at diagnosis [*n* (%)]	
Grade II	88 (51.1)
Grade III	55 (32.0)
Grade IV	29 (16.9)
Refined Minnesota aGVHD risk score at aGVHD diagnosis [*n* (%)]	
Standard risk	116 (67.4)
High risk	56 (32.6)
Severity of skin aGVHD before xenopax treatment [*n* (%)]	
Stage 0	98 (57.6)
Stage 1	17 (9.9)
Stage 2	33 (19.2)
Stage 3	21 (12.2)

Characteristics of aGVHD	Summary statistics
Stage 4	2 (1.3)
Severity of gut aGVHD before xenopax treatment [*n* (%)]	
Stage 0	44 (25.5)
Stage 1	20 (11.6)
Stage 2	43 (25.0)
Stage 3	33 (19.2)
Stage 4	32 (18.6)
Severity of liver aGVHD before xenopax treatment [*n* (%)]	
Stage 0	117 (68.1)
Stage 1	8 (4.7)
Stage 2	29 (16.9)
Stage 3	16 (9.3)
Stage 4	2 (1.2)
Overall severity of aGVHD before xenopax treatment [*n* (%)]	
Grade II	76 (44.2)
Grade III	62 (36.0)
Grade IV	34 (19.8)
Refined Minnesota aGVHD risk score before xenopax treatment [*n* (%)]	
Standard risk	105 (61.0)
High risk	67 (39.0)

aGVHD acute graft-versus-host disease

Table S5 Overall response at different times after xenopax treatment between different groups [*n* (%, 95%CI)]

**Response assessments**	**Total patients (*n* = 172)**		**Type of xenopax treatment**					**Line of xenopax treatment**				
			**Monotherapy (*n* = 60)**		**Combined therapy (*n* = 112)**			**Second-line (*n* = 60)**		**≥ Second-line (*n* = 112)**		
	***n* (%)**	**95%CI**	***n* (%)**	**95%CI**	***n* (%)**	**95%CI**	***P*-value**	***n* (%)**	**95%CI**	***n* (%)**	**95%CI**	***P*-value**
Response rate												
CR	104 (60.5)	53.2 – 67.8	35 (58.3)	45.9 – 70.8	69 (61.6)	52.6 – 70.6	0.676	35 (58.3)	45.9 – 70.8	69 (61.6)	52.6 – 70.6	0.676
PR	38 (22.1)	15.9 – 28.3	13 (21.7)	11.2 – 32.1	25 (22.3)	14.6 – 30.0	0.921	10 (16.7)	7.2 – 26.1	28 (25.0)	17.0 – 33.0	0.209
Overall ORR	142 (82.6)	76.9 – 88.2	48 (80.0)	69.9 – 90.1	94 (83.9)	77.1 – 90.7	0.518	45 (75.0)	64.0 – 86.0	97 (86.6)	80.3 – 92.9	0.056
ORR at day 28	111 (64.5)	57.4 – 71.7	39 (65.0)	52.9 – 77.1	72 (64.3)	55.4 – 73.2	0.926	37 (61.7)	49.4 – 74.0	74 (66.1)	57.3 – 74.8	0.565
ORR at day 42	119 (69.2)	62.3 – 76.1	41 (68.3)	56.6 – 80.1	78 (69.6)	61.1 – 78.2	0.859	40 (66.7)	54.7 – 78.6	79 (70.5)	62.1 – 79.0	0.600
ORR at day 56	131 (76.2)	69.8 – 82.5	46 (76.7)	66.0 – 87.4	85 (75.9)	68.0 – 83.8	0.910	44 (73.3)	62.1 – 84.5	87 (77.7)	70.0 – 85.4	0.524

ORR was defined as CR plus PR. *CR* complete response, *PR* partial response, *ORR* overall response rate

Table S6 Subgroup analysis for overall response rate (ORR) at day 28 and at any time after xenopax treatment

**Overall response to treatment/Patients evaluable**	**At day 28 (*n* = 172)**		**At any time (*n* = 172)**	
	***n* (%)**	**95% CI**	***n* (%)**	**95% CI**
Age				
Children (< 18 years)	41 (67.2)	55.4 – 79.0	54 (88.5)	80.5 – 96.5
Adults (≥ 18 years)	70 (63.0)	54.1 – 72.0	88 (79.3)	71.7 – 86.8
18 − 50 years	47 (64.4)	53.4 – 75.4	59 (80.8)	71.8 – 89.9
≥ 50 years	23 (60.5)	45.0 – 76.1	29 (76.3)	62.8 – 89.8
Gender				
Male	68 (67.3)	58.2 – 76.5	85 (84.2)	77.0 – 91.3
Female	43 (60.6)	49.2 – 71.9	57 (80.3)	71.0 – 89.5
Underlying disease				
Hematologic malignancies	93 (62.4)	54.6 – 70.2	120 (80.5)	74.2 – 86.9
Non-hematologic malignancies	18 (78.2)	61.4 – 95.1	22 (95.7)	78.1 – 99.9
Donor-recipient gender matched				
Female to male	16 (51.6)	34.0 – 69.2	23 (74.2)	58.8 – 89.6
Others	95 (67.4)	59.6 – 75.1	119 (84.4)	78.4 – 90.4
Donor-recipient relation				
Mother-child	6 (60.0)	29.6 – 90.4	8 (80.0)	44.4 – 97.5
Others	105 (64.8)	57.5 – 72.2	134 (82.7)	76.9 – 88.5
Donor type				
Matched sibling donor	23 (59.0)	43.5 – 74.4	29 (74.4)	60.7 – 88.1
Haploidentical related donor	70 (66.0)	57.0 – 75.1	91 (85.8)	79.2 – 92.5
Unrelated donor	8 (61.5)	35.1 – 88.0	9 (69.2)	44.1 – 94.3
Umbilical cord blood	10 (71.4)	47.8 – 95.1	13 (92.9)	66.1 – 99.8
Number of other second-line treatments before xenopax				
0 type	37 (61.7)	49.4 – 74.0	45 (75.0)	64.0 – 86.0
1 type	41 (68.3)	56.6 – 80.1	50 (83.3)	73.9 – 92.8
≥ 2 types	33 (63.5)	50.4 – 76.5	47 (90.4)	82.4 – 98.4
Refined Minnesota aGVHD risk score before xenopax treatment				

Overall response to treatment/Patients evaluable	At day 28 (*n* = 172)		At any time (*n* = 172)	
	n **(%)**	**95% CI**	***n*** **(%)**	**95% CI**
Standard risk	77 (73.3)	64.9 – 81.8	90 (85.7)	79.0 – 92.4
High risk	34 (50.8)	38.8 – 62.7	52 (77.7)	67.6 – 87.6
Severity of aGVHD before xenopax treatment				
Grade II	57 (75.0)	65.3 – 84.7	64 (84.2)	76.0 – 92.4
Grade III	42 (67.7)	56.1 – 79.4	54 (87.1)	78.8 – 95.4
Grade IV	12 (35.3)	19.2 – 51.4	24 (70.6)	55.3 – 85.9
Organ involvement before xenopax treatment				
Skin aGVHD	47 (63.5)	52.5 – 74.5	63 (85.1)	77.0 – 93.2
Gut aGVHD	75 (58.1)	49.6 – 66.7	103 (79.8)	72.9 – 86.8
Liver aGVHD	35 (62.5)	49.8 – 75.2	43 (76.8)	65.7 – 87.8
Number of organs involved before xenopax treatment				
1	67 (69.1)	59.9 – 78.3	81 (83.5)	76.1 – 90.9
2	40 (65.6)	53.7 – 77.5	53 (86.9)	78.4 – 95.4
3	4 (28.6)	4.9 – 52.2	8 (57.1)	31.2 – 83.1
Initial dose of steroid				
Methylprednisolone ≥ 2 mg/(kg·d)	40 (57.1)	45.5 – 68.7	59 (84.3)	75.8 – 92.8
Methylprednisolone < 2 mg/(kg·d)	71 (69.6)	60.7 – 78.5	83 (81.4)	73.8 – 88.9
Type of steroid refractory before xenopax treatment				
Steroid-resistant aGVHD	93 (65.0)	57.2 – 72.9	119 (83.2)	77.1 – 89.3
Steroid-dependent aGVHD	18 (62.1)	44.4 – 79.7	23 (79.3)	64.6 – 94.1
Patients had active infections when using xenopax				
No	64 (61.0)	51.6 – 70.3	82 (78.1)	70.2 – 86.0
Yes	47 (70.1)	59.2 – 81.1	60 (89.6)	82.2 – 96.9

aGVHD acute graft-versus-host disease

Table S7 Overall response rate, OS, and NRM in different subgroups after xenopax treatment

Response and outcomes	Conditioning regimen			Donor type		
	**Chemotherapy-based regimen (*n* = 151)**	**Total body irradiation-based regimen (*n* = 21)**	***P*-value**	**Matched sibling donor (*n* = 39)**	**Alternative donor (*n* = 133)**	***P*-value**
Response rate						
ORR at day 28			0.233			0.409
n (%)	95 (62.9)	16 (76.2)		23 (59.0)	88 (66.2)	
95% CI	55.2 – 70.6	58.0 – 94.4		43.6 – 74.4	58.2 – 74.2	
ORR at any time			0.684			0.125
n (%)	124 (82.1)	18 (85.7)		29 (74.4)	113 (85.0)	
95% CI	76.0 – 88.2	70.7 – 100.0		60.7 – 88.1	78.9 – 91.1	
2-year probability of OS [%, (95% CI)]	67.5 (60.1 – 74.9)	71.4 (52.0 – 90.8)	0.849	53.8 (38.1 – 69.5)	72.2 (64.6 – 79.8)	0.027
2-year cumulative incidence of NRM [%, (95% CI)]	26.6 (19.3 – 33.9)	24.6 (5.8 – 43.4)	0.941	38.1 (22.4 – 53.8)	23.1 (15.8 – 30.4)	0.069

ORR overall response rate, *OS* overall survival, *NRM* non-relapse mortality

Table S8 Patient characteristics between monotherapy and combined therapy groups

**Variables**	**Monotherapy (*n* = 60)**	**Combined therapy (*n* = 112)**	**Z/χ^{2*}**	***P*-value**
Age at HSCT [years, median (range)]	37 (1 – 61)	19 (1 – 74)	– 3.818	< 0.001
Female sex [*n* (%)]	26 (43.3)	45 (40.2)	0.160	0.689
Underlying disease [*n* (%)]			0.610	0.435
Hematologic malignancies	54 (90.0)	95 (84.8)		
HCT-CI score [*n* (%)]			4.386	0.112
Low risk	39 (92.9)	107 (82.3)		
Intermediate risk	2 (4.8)	21 (16.2)		
High risk	1 (2.4)	2 (1.5)		
Donor–recipient relationship [*n* (%)]			0.111	0.738
Mother–child	3 (5.0)	7 (6.3)		
Others	57 (95.0)	105 (93.8)		
Donor-recipient sex matched [*n* (%)]			0.828	0.363
Female to male	13 (21.7)	18 (16.1)		
Others	47 (78.3)	94 (83.9)		
Donor type [*n* (%)]			0.284	0.594
Matched sibling donor	15 (25.0)	24 (21.4)		
Alternative donor	45 (75.0)	88 (78.6)		
Conditioning regimen [*n* (%)]			0.109	0.742
Chemotherapy-based regimen	52 (86.7)	99 (88.4)		
Total body irradiation-based regimen	8 (13.3)	13 (11.6)		
Time from diagnosis of aGVHD to xenopax treatment [d, median (range)]	5 (0 – 69)	6 (0 – 161)	– 1.044	0.297
Overall severity of aGVHD before xenopax treatment [*n* (%)]			9.334	0.002
Grade II	36 (60.0)	40 (35.7)		
Grade III – IV	24 (40.0)	72 (64.3)		
Refined Minnesota aGVHD risk score before xenopax treatment [*n* (%)]			2.057	0.151
Standard risk	41 (68.3)	64 (57.1)		
High risk	19 (31.7)	48 (42.9)		
Steroid refractory type [*n* (%)]			2.309	0.315
Steroid resistant	51 (85.0)	92 (82.1)		
Steroid dependent	9 (15.0)	20 (17.9)		

*χ^2 value for categorical data and *Z* value for continuous data. *aGVHD* acute graft-versus-host disease, *HCT-CI* hematopoietic cell transplantation-comorbidity index, *HSCT* hematopoietic stem cell transplantation

Table S9 New onset infections after xenopax treatment in subgroup analysis [*n* (%)]

**Infection categories**	**Line of xenopax treatment**			**Type of xenopax treatment**			**Severity of aGVHD**		
	**Second line (*n* = 60)**	**≥ Second line (*n* = 112)**	***P*-value**	**Monotherapy (*n* = 60)**	**Combined therapy (*n* = 112)**	***P*-value**	**Grade II aGVHD (*n* = 76)**	**Grade III – IV aGVHD (*n* = 96)**	***P*-value**
Viral infection	9 (15.0)	31 (27.7)	0.061	5 (8.3)	35 (31.3)	0.001	11 (14.5)	29 (30.2)	0.015
Bacterial infection	7 (11.7)	21 (18.8)	0.230	2 (3.3)	26 (23.2)	0.001	10 (13.2)	18 (18.8)	0.324
Fungal infection	3 (5.0)	7 (6.3)	0.738	1 (1.7)	9 (8.0)	0.089	4 (5.3)	6 (6.3)	0.784
Any infection (≥ 1 type)	22 (36.7)	43 (38.4)	0.824	16 (26.7)	49 (43.8)	0.028	19 (25.0)	46 (47.9)	0.002
Multiple infection (≥ 2 types)	5 (8.3)	17 (15.2)	0.200	3 (5.0)	19 (17.0)	0.025	9 (11.8)	13 (13.5)	0.740

aGVHD acute graft-versus-host disease

Table S10 Characteristics of cGVHD ($n = 30$)

**Characteristics of cGVHD**	**Summary statistics**
Time from the beginning of xenopax treatment to cGVHD [d, median (range)]	120 (7 – 555)
Grade of cGVHD [*n* (%)]	
Mild	21 (70.0)
Moderate	8 (26.7)
Severe	1 (3.3)
Site of cGVHD [*n* (%)]	
Skin	21 (70.0)
Mouth	7 (23.3)
Eye	2 (6.7)
Gut	10 (33.3)
Liver	8 (26.7)
Lung	5 (16.7)
Others	4 (13.3)
Number of sites [*n* (%)]	
1	11 (36.7)
2	12 (40.0)
≥ 3	7 (23.3)

cGVHD chronic graft-versus-host disease

Table S11 Clinical outcomes at 2 years after xenopax treatment in subgroup analysis [cumulative incidence, % (95% CI)]

**Patient subgroups**	**Relapse**		**NRM**		**DFS**		**OS**	
	**Cumulative incidence**	***P*-value**	**Cumulative incidence**	***P*-value**	**Cumulative incidence**	***P*-value**	**Cumulative incidence**	***P*-value**
Severity of aGVHD before xenopax treatment		0.004		0.952		0.023		0.428
Grade II	28.6 (17.9 − 39.3)		23.7 (14.0 − 33.3)		48.2 (38.0 − 61.0)		65.8 (55.9 − 77.4)	
Grade III − IV	11.5 (4.4 − 18.6)		25.0 (16.3 − 33.7)		63.9 (54.8 − 74.5)		69.8 (61.2 − 79.6)	
Refined Minnesota aGVHD risk score before xenopax treatment		0.005		0.004		0.685		0.101
Standard risk	26.4 (17.3 − 35.5)		17.1 (9.9 − 24.4)		57.3 (48.4 − 67.8)		72.4 (64.3 − 81.5)	
High risk	7.5 (1.1 − 13.8)		35.8 (24.2 − 47.4)		56.6 (45.9 − 69.8)		61.2 (50.6 − 74.1)	
Type of xenopax treatment		0.003		0.812		0.003		0.328
Monotherapy	30.1 (18.3 − 41.8)		23.3 (12.5 − 34.1)		46.5 (35.4 − 61.1)		63.3 (52.2 − 76.8)	
Combined therapy	13.5 (6.2 − 20.9)		25.0 (16.9 − 33.1)		62.4 (53.8 − 72.4)		70.5 (62.6 − 79.5)	
Line of xenopax treatment*		0.931		0.211		0.222		0.180
Second line	18.4 (8.5 − 28.3)		30.0 (18.3 − 41.7)		51.6 (40.4 − 66.0)		61.7 (50.5 − 75.3)	
≥ Second line	19.4 (11.4 − 27.5)		21.4 (13.8 − 29.1)		59.7 (51.1 − 69.9)		71.4 (63.5 − 80.3)	

*Second line denotes xenopax as the first second-line therapy for aGVHD after first-line treatment failure; ≥ second line indicates xenopax used in third-line or higher-order therapies following second-line treatment failure/intolerance. *aGVHD* acute graft-versus-host disease, *CI* confidence interval, *NRM* non-relapse mortality, *DFS* disease-free survival, *OS* overall survival

Table S12 Causes of death

**Causes of death**	***n* (%)**
Relapse	14 (8.1)
NRM	
Infection	18 (10.5)
Graft-versus-host disease	8 (4.7)
Thrombotic microangiopathy	2 (1.2)
Gastrointestinal hemorrhage	1 (0.6)
Heart failure	2 (1.2)
Intracerebral hemorrhage	5 (2.9)
Others	5 (2.9)

NRM non-relapse mortality

Table S13 Patient characteristics between best available treatments (BATs) and xenopax groups

**Variables**	**BATs (*n* = 1009)**	**Xenopax (*n* = 172)**	***Z/χ²****	***P*-value**
Age [years, median (range)]	24 (1 − 74)	30 (1 − 74)	-2.293	0.022
Female sex [*n* (%)]	410 (40.6)	71 (41.3)	0.025	0.874
Underlying disease [*n* (%)]			5.787	0.055
Hematologic malignancies	847 (83.9)	147 (85.5)		
Severe aplastic anemia	132 (13.1)	15 (8.7)		
Others	30 (3.0)	10 (5.8)		
Donor type [*n* (%)]			7.086	0.008
Matched sibling donor	148 (14.7)	39 (22.7)		
Alternative donor	861 (85.3)	133 (77.3)		
Overall severity of aGVHD before treatment [*n* (%)]			2.683	0.101
Grade II	514 (50.9)	76 (44.2)		
Grade III − IV	495 (49.1)	96 (55.8)		

*χ^2 value for categorical data using χ^2 tests and Fisher's exact tests and Z value for continuous data using Student's t test. *aGVHD* acute graft-versus-host disease

Table S14 Patient characteristics between basiliximab and xenopax groups

Variables	Basiliximab (n = 940)	Xenopax (n = 172)	Z/χ^{2*}	P-value
Age [years, median (range)]	24 (1 − 63)	30 (1 − 74)	−2.571	0.010
Female sex [n (%)]	382 (40.6)	71 (41.3)	0.025	0.875
Underlying disease [n (%)]			6.099	0.047
Hematologic malignancies	785 (83.5)	147 (85.5)		
Severe aplastic anemia	127 (13.5)	15 (8.7)		
Others	28 (3.0)	10 (5.8)		
Donor type [n (%)]			19.258	< 0.001
Matched sibling donor	100 (10.6)	39 (22.7)		
Alternative donor	840 (89.4)	133 (77.3)		
Overall severity of aGVHD before treatment [n (%)]			3.475	0.062
Grade II	488 (51.9)	76 (44.2)		
Grade III − IV	452 (48.1)	96 (55.8)		

[*]χ^2 value for categorical data using χ^2 tests and Fisher's exact tests and Z value for continuous data using Student's t test. *aGVHD* acute graft-versus-host disease

Table S15 Patient characteristics between mesenchymal stromal cells (MSCs) and xenopax groups

**Variables**	**MSCs (*n* = 14)**	**Xenopax (*n* = 172)**	***Z*/χ^{2*}**	***P*-value**
Age [years, median (range)]	19 (6−54)	30 (1−74)	-1.291	0.197
Female sex [*n* (%)]	8 (57.1)	71 (41.3)	1.333	0.248
Underlying disease [*n* (%)]			2.661	0.264
Hematologic malignancies	12 (85.7)	147 (85.5)		
Severe aplastic anemia	0	15 (8.7)		
Others	2 (14.3)	10 (5.8)		
Donor type [*n* (%)]			4.017	0.045
Matched sibling donor	0	39 (22.7)		
Alternative donor	14 (100.0)	133 (77.3)		
Overall severity of aGVHD before treatment [*n* (%)]			1.288	0.256
Grade II	4 (28.6)	76 (44.2)		
Grade III − IV	10 (71.4)	96 (55.8)		

[*]χ^2 value for categorical data using χ^2 tests and Fisher's exact tests and Z value for continuous data using Student's t test.
aGVHD acute graft-versus-host disease

Table S16 Patient characteristics between mesenchymal stromal cells (MSCs) plus basiliximab and xenopax groups

**Variables**	**MSCs plus basiliximab (*n* = 40)**	**Xenopax (*n* = 172)**	Z/χ^{2*}	***P*-value**
Age [years, median (range)]	38 (17 − 62)	30 (1 − 74)	−2.726	0.006
Female sex [*n* (%)]	15 (37.5)	71 (41.3)	0.192	0.661
Underlying disease [*n* (%)]			4.525	0.104
Hematologic malignancies	39 (97.5)	147 (85.5)		
Severe aplastic anemia	1 (2.5)	15 (8.7)		
Others	0	10 (5.8)		
Donor type [*n* (%)]			6.562	0.010
Matched sibling donor	17 (42.5)	39 (22.7)		
Alternative donor	23 (57.5)	133 (77.3)		
Overall severity of aGVHD before treatment [*n* (%)]			0.904	0.342
Grade II	21 (52.5)	76 (44.2)		
Grade III − IV	19 (47.5)	96 (55.8)		

$^{*}\chi^2$ value for categorical data using χ^2 tests and Fisher's exact tests and Z value for continuous data using Student's t test.
aGVHD acute graft-versus-host disease

Table S17 Patient characteristics between ruxolitinib and xenopax groups

Variables	Ruxolitinib (*n* = 15)	Xenopax (*n* = 172)	Z/χ^{2*}	*P*-value
Age [years, median (range)]	20 (5 − 48)	30 (1 − 74)	-1.137	0.256
Female sex [*n* (%)]	5 (33.3)	71 (41.3)	0.361	0.548
Underlying disease [*n* (%)]			5.486	0.064
Hematologic malignancies	11 (73.3)	147 (85.5)		
Severe aplastic anemia	4 (26.7)	15 (8.7)		
Others	0	10 (5.8)		
Donor type [*n* (%)]			4.297	0.038
Matched sibling donor	0	39 (22.7)		
Alternative donor	15 (100.0)	133 (77.3)		
Overall severity of aGVHD before treatment [*n* (%)]			8.018	0.005
Grade II	1 (6.7)	76 (44.2)		
Grade III − IV	14 (93.3)	96 (55.8)		

[*] χ^2 value for categorical data using χ^2 tests and Fisher's exact tests and Z value for continuous data using Student's t test.
aGVHD acute graft-versus-host disease

Table S18 The comparison of enrollment time, second-line treatment for SR-aGVHD, infection rate, OS and NRM between ruxolitinb (REACH1) and xenopax

References	Treatment	Time	Rate of at least 1 infection (%)	ORR at day 28 (%)	ORR at any time (%)	OS [%, (95% CI)]	NRM [%, (95%CI)]
Jagasia et al. [9]	Ruxolitinib	27 November 2016 to 2 July 2018	80.3	55.0	73.2	1-year: 42.6 (30.0 – 54.6)	1-year: 52.9 (39.6 – 64.5)
This study	Xenopax	January 1, 2020 to October 31, 2023	37.8	64.5	82.6	2-year: 68.0 (61.4 – 75.4)	2-year: 24.2 (18.0 – 30.9)

OS overall survival, *NRM* non-relapse mortality, *SR-aGVHD* steroid refractory acute graft-versus-host disease

Table S19 Univariate and multivariate analysis for response and clinical outcomes in total cohort with steroid-refractory acute graft-versus-host disease after second-line treatments

**Covariate**	**Univariate analysis**			**Multivariate analysis**		
	HR	**95% CI**	***P*-value**	***HR***	**95% CI**	***P*-value**
Lack of response at day 28						
Age			0.019			
< 18 years		1				
≥ 18 years	1.372	1.052 – 1.788				
Donor type			0.035			
Matched sibling donor		1				
Others	1.333	1.020 – 1.741				
Severity of aGVHD before second-line treatment			< 0.001			< 0.001
Grade II		1			1	
Grade III – IV	2.823	2.176 – 3.663		2.823	2.176 – 3.663	
Lack of response at any time						
Age			< 0.001			
< 18 years		1				
≥ 18 years	1.745	1.282 – 2.376				
HCI-CI score			0.055			
0 score		1				
≥ 1 score	1.533	0.990 – 2.372				
Severity of aGVHD before second-line treatment			< 0.001			
Grade II		1				
Grade III – IV	2.053	1.543 – 2.730				
Infection						
Treatment			< 0.001			< 0.001
Xenopax		1			1	
BATs	2.548	1.826 – 3.556		2.663	1.806 – 3.927	
HCI-CI score			0.004			
0 score		1				
≥1 score	1.714	1.193 – 2.463				
Severity of aGVHD before second-line treatment			0.020			0.004
Grade II		1			1	
Grade III – IV	1.315	1.043 – 1.657		1.739	1.190 – 2.541	
Treatment failure as defined by OS						
Age			< 0.001			0.001

Covariate	Univariate analysis			Multivariate analysis		
	HR	95% CI	*P*-value	*HR*	95% CI	*P*-value
< 18 years		1			1	
≥ 18 years	2.146	1.705 – 2.702		1.822	1.269 – 2.616	
HCT-CI score			< 0.001			0.001
0 score		1			1	
≥ 1 score	1.812	1.347 – 2.437		1.666	1.220 – 2.275	
Severity of aGVHD before second-line treatment			< 0.001			0.008
Grade II		1			1	
Grade III – IV	2.011	1.646 – 2.457		1.510	1.111 – 2.052	
NRM						
Age			< 0.001			0.001
< 18 years		1			1	
≥ 18 years	2.146	1.705 – 2.702		1.822	1.269 – 2.616	
HCT-CI score			< 0.001			0.001
0 score		1			1	
≥ 1 score	1.812	1.347 – 2.437		1.666	1.220 – 2.275	
Severity of aGVHD before second-line treatment			< 0.001			0.001
Grade II		1			1	
Grade III – IV	2.011	1.646 – 2.457		1.510	1.111 – 2.052	

OS overall survival, *BATs* best available treatments, *NRM* non-relapse mortality, *HCT-CI* hematopoietic cell transplantation-comorbidity index, *aGVHD* acute graft-versus-host disease, *HR* hazard ratio, *CI* confidence interval

Table S20 The cost of different drugs during treatment of acute GVHD

**Drugs**	**ORR at any time (%)**	**Median effective number of doses**	**Median effective time of treatment (d)**	**Unit price (RMB)***	**Total drug cost (RMB)**	**Cost per ORR (RMB)**
Basiliximab	78.6	4	21	7966	31,864	40,539
MSCs	57.1	2	14	12,000	24,000	42,031
Ruxolitinib	62.5	20	10	98	1960	3136
Xenopax	82.6	3	17	8750	26,250	31,780

*It was calculated according to the standard of the patient's body weight of 60 kg. *ORR* overall response rate, *MSCs* mesenchymal stromal cells

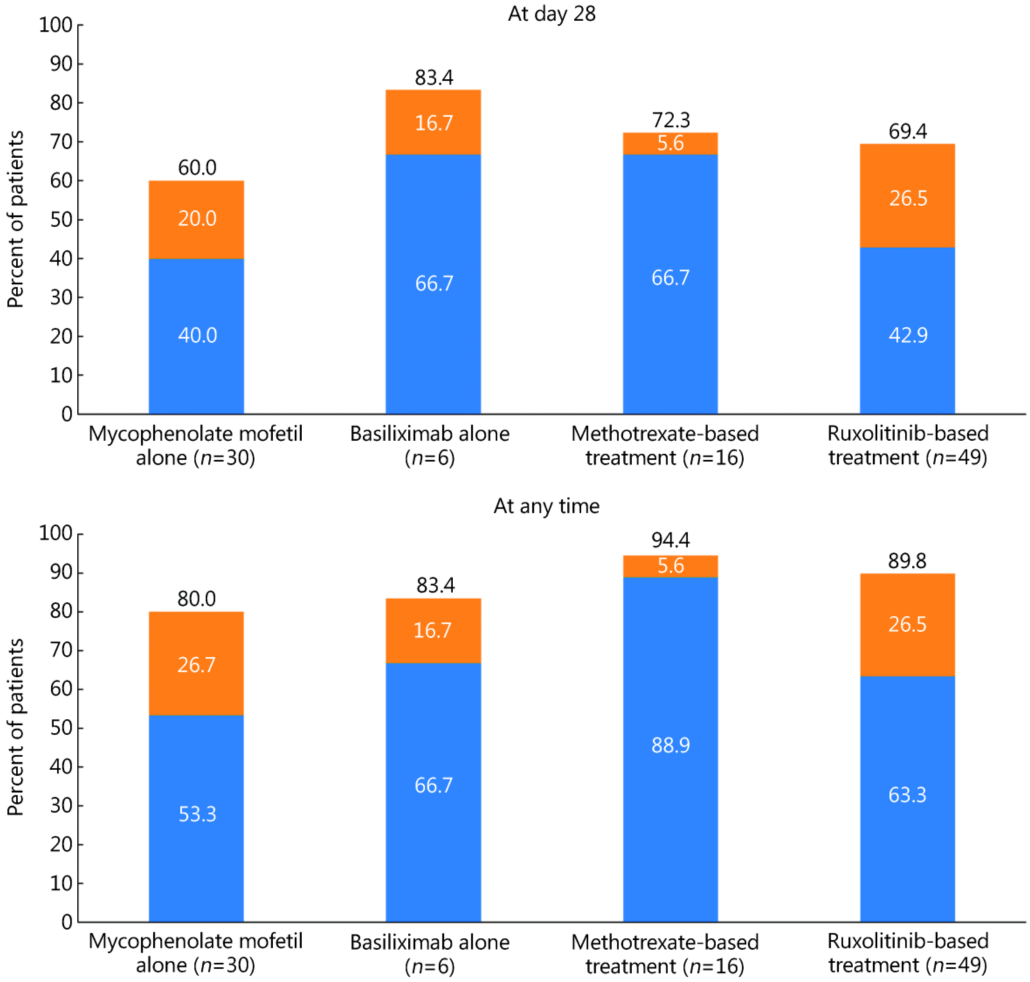

Fig. S1 Overall response of patients receiving other second-line treatments before xenopax at day 28 and at any time. Overall response included complete response (blue) plus partial response (orange) rates

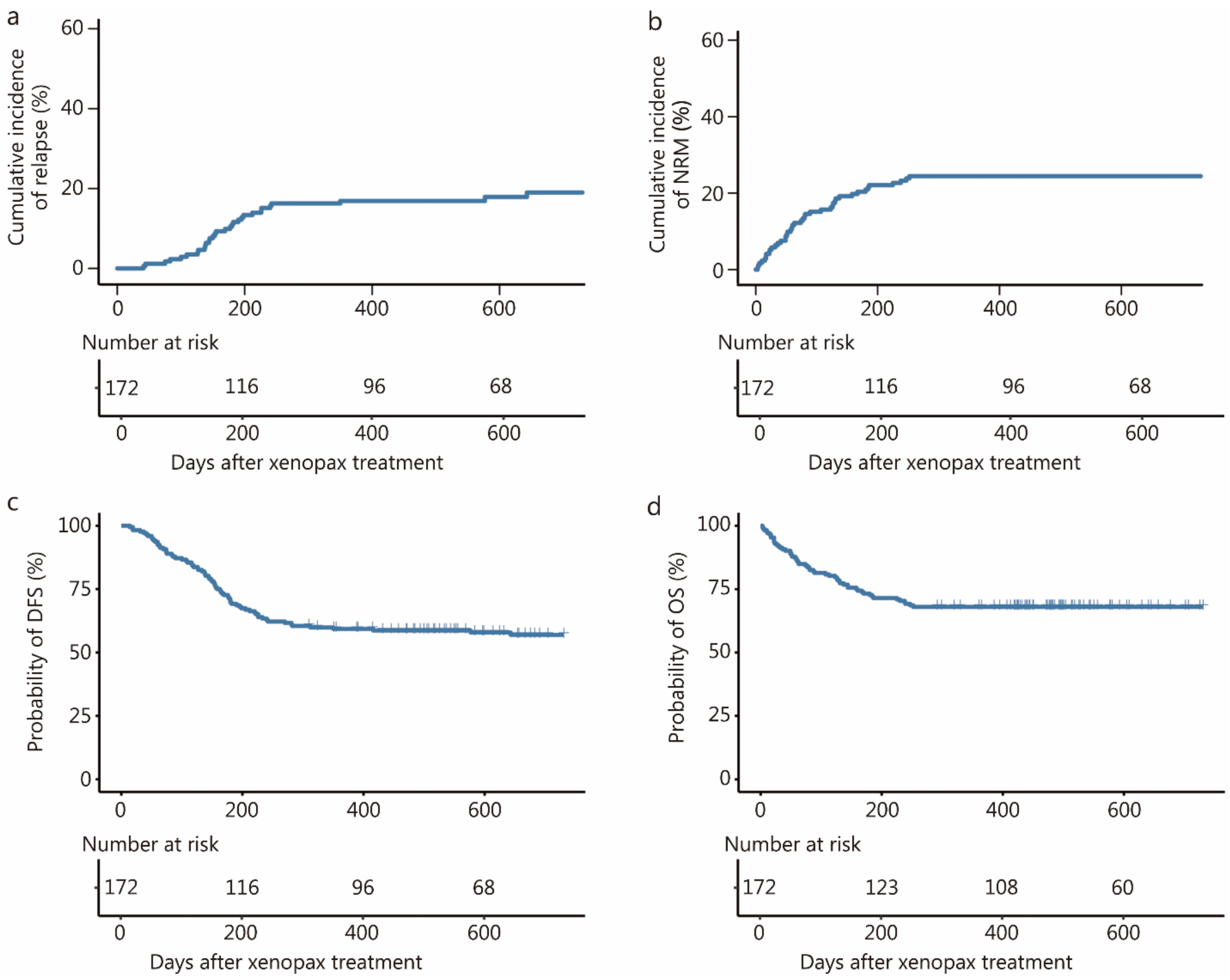

Fig. S2 Clinical outcomes at 2 years after xenopax treatment. **a** Cumulative incidence of relapse. **b** Cumulative incidence of NRM. **c** Probability of DFS. **d** Probability of OS. Cumulative of relapse and NRM curves depict the cumulative proportion of patients experiencing events over time, exhibiting a monotonically non-decreasing trend; DFS and OS curves represent the proportion of patients surviving over time, showing a monotonically non-increasing trend, collectively characterizing the dynamic features of survival processes and event risks. NRM non-relapse mortality, DFS disease-free survival, OS overall survival

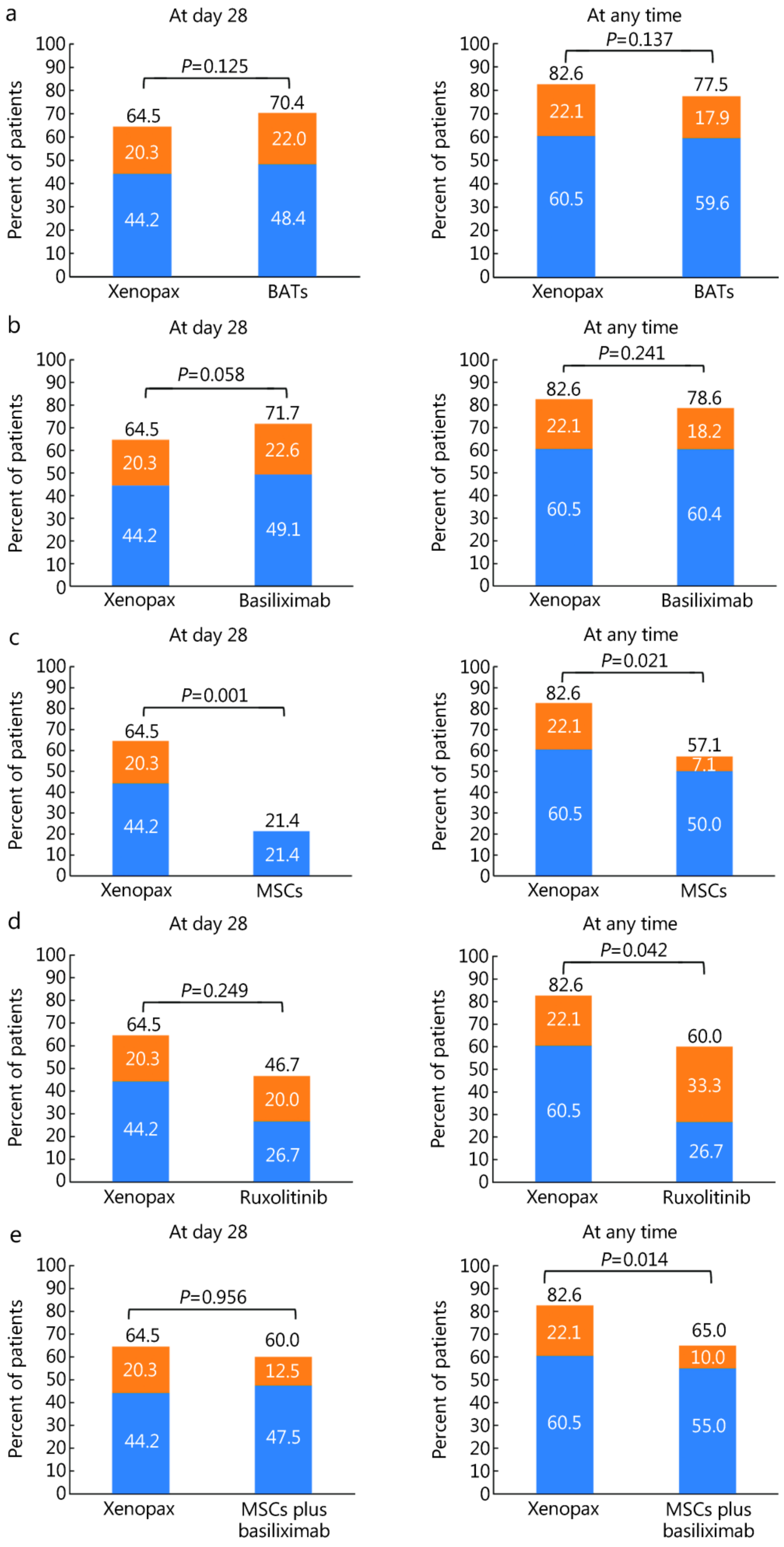
a
At day 28
Percent of patients
P=0.125
64.5
20.3
44.2
70.4
22.0
48.4
Xenopax
BATs
At any time
P=0.137
82.6
22.1
60.5
77.5
17.9
59.6
Xenopax
BATs
b
At day 28
P=0.058
64.5
20.3
44.2
71.7
22.6
49.1
Xenopax
Basiliximab
At any time
P=0.241
82.6
22.1
60.5
78.6
18.2
60.4
Xenopax
Basiliximab
c
At day 28
P=0.001
64.5
20.3
44.2
21.4
21.4
Xenopax
MSCs
At any time
P=0.021
82.6
22.1
60.5
57.1
7.1
50.0
Xenopax
MSCs
d
At day 28
P=0.249
64.5
20.3
44.2
46.7
20.0
26.7
Xenopax
Ruxolitinib
At any time
P=0.042
82.6
22.1
60.5
60.0
33.3
26.7
Xenopax
Ruxolitinib
e
At day 28
P=0.956
64.5
20.3
44.2
60.0
12.5
47.5
Xenopax
MSCs plus basiliximab
At any time
P=0.014
82.6
22.1
60.5
65.0
10.0
55.0
Xenopax
MSCs plus basiliximab

Fig. S3 The overall response at day 28 and at any time of (**a**) xenopax vs. best available treatments (BATs), (**b**) xenopax vs. basiliximab, (**c**) xenopax vs. mesenchymal stromal cells (MSCs), (**d**) xenopax vs. ruxolitinib, and (**e**) xenopax vs. MSCs plus basiliximab. Overall response included complete response (blue) plus partial response (orange) rates

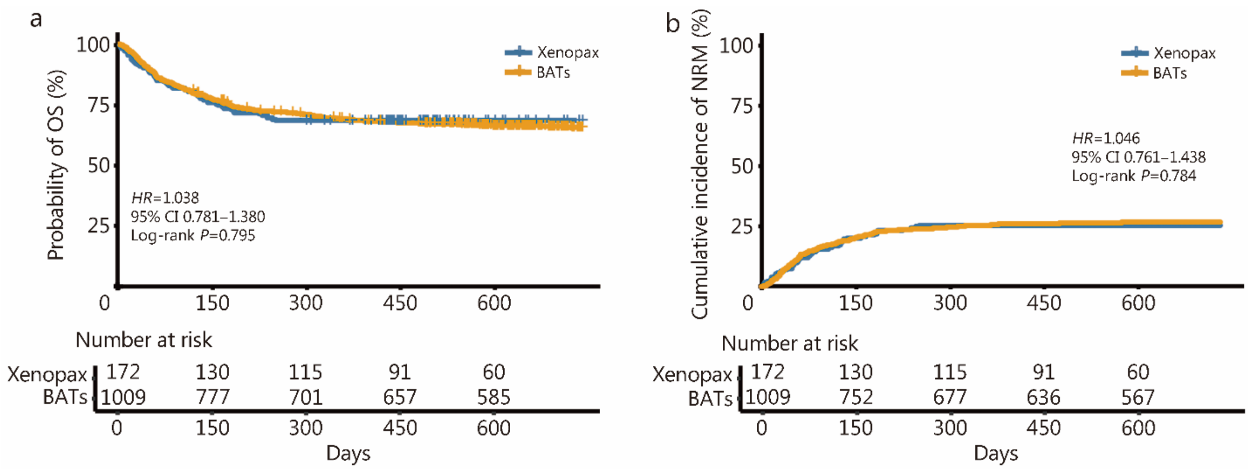

Fig. S4 Clinical outcomes at 2 years after xenopax and best available treatments (BATs) treatment. **a** Probability of OS. **b** Cumulative incidence of NRM. Cumulative of NRM curve depicts the cumulative proportion of patients experiencing events over time, exhibiting a monotonically non-decreasing trend; OS curve represents the proportion of patients surviving over time, showing a monotonically non-increasing trend. OS overall survival, NRM non-relapse mortality

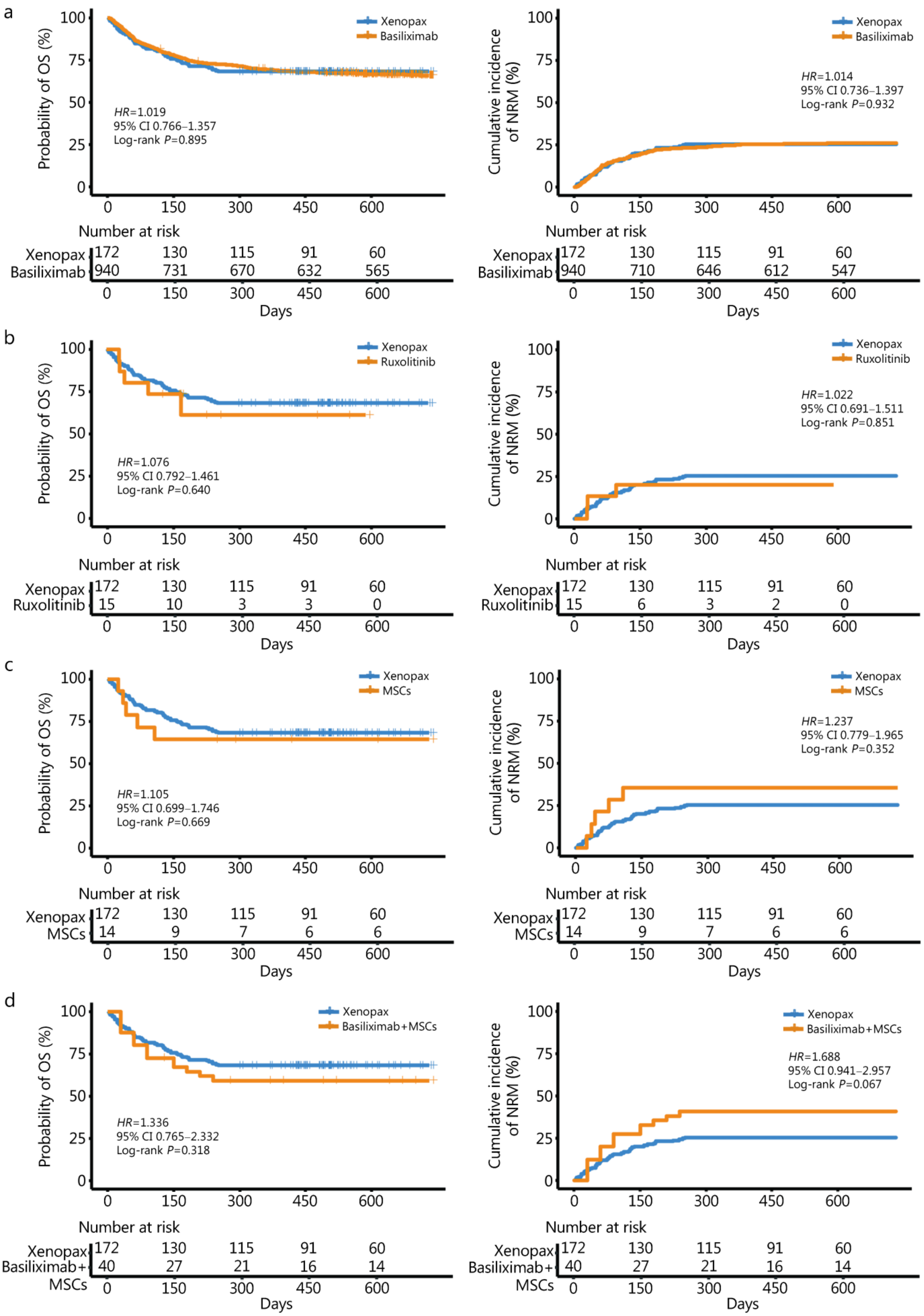
a
Probability of OS (%)
Xenopax
Basiliximab
HR=1.019
95% CI 0.766–1.357
Log-rank P=0.895
Number at risk
Xenopax 172 130 115 91 60
Basiliximab 940 731 670 632 565
Days
Cumulative incidence of NRM (%)
HR=1.014
95% CI 0.736–1.397
Log-rank P=0.932
Xenopax 172 130 115 91 60
Basiliximab 940 710 646 612 547
b
Ruxolitinib
HR=1.076
95% CI 0.792–1.461
Log-rank P=0.640
Xenopax 172 130 115 91 60
Ruxolitinib 15 10 3 3 0
HR=1.022
95% CI 0.691–1.511
Log-rank P=0.851
Xenopax 172 130 115 91 60
Ruxolitinib 15 6 3 2 0
c
MSCs
HR=1.105
95% CI 0.699–1.746
Log-rank P=0.669
Xenopax 172 130 115 91 60
MSCs 14 9 7 6 6
HR=1.237
95% CI 0.779–1.965
Log-rank P=0.352
Xenopax 172 130 115 91 60
MSCs 14 9 7 6 6
d
Basiliximab+MSCs
HR=1.336
95% CI 0.765–2.332
Log-rank P=0.318
Xenopax 172 130 115 91 60
Basiliximab+MSCs 40 27 21 16 14
HR=1.688
95% CI 0.941–2.957
Log-rank P=0.067
Xenopax 172 130 115 91 60
Basiliximab+MSCs 40 27 21 16 14
0 150 300 450 600
0 25 50 75 100

Fig. S5 Clinical outcomes at 2 years after xenopax and best available treatments (BATs). Probability of OS and cumulative incidence of NRM of (**a**) xenopax vs. basiliximab, (**b**) xenopax vs. ruxolitinib, (**c**) xenopax vs. mesenchymal stromal cells (MSCs), and (**d**) xenopax vs. basiliximab plus MSCs. Cumulative of NRM curve depicts the cumulative proportion of patients experiencing events over time, exhibiting a monotonically non-decreasing trend; OS curve represents the proportion of patients surviving over time, showing a monotonically non-increasing trend. OS overall survival, NRM non-relapse mortality

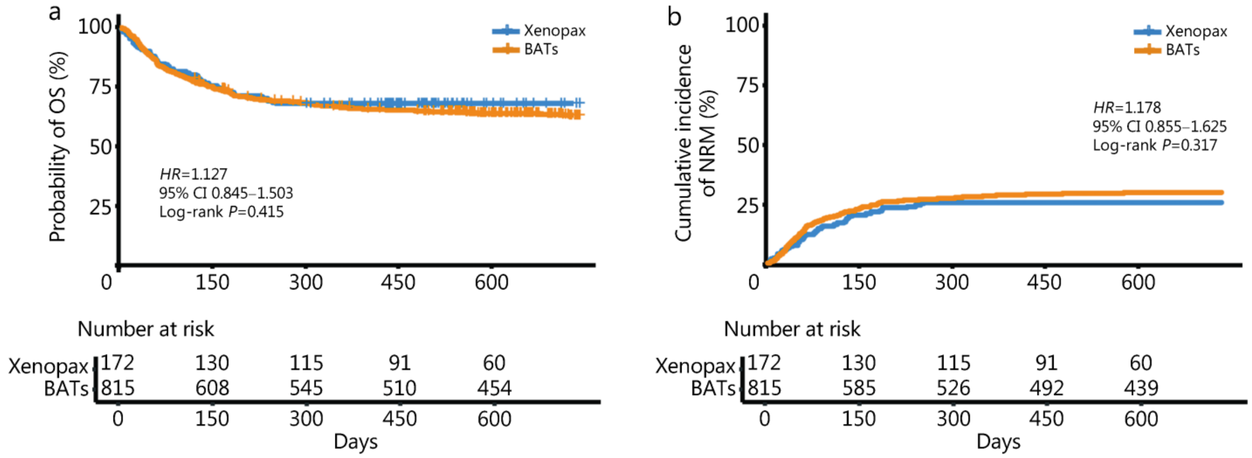

Fig. S6 Clinical outcomes at 2 years between xenopax and best available treatments (BATs) groups in the propensity score matching (PSM) analysis. **a** Probability of OS. **b** Cumulative incidence of NRM. Cumulative of NRM curve depicts the cumulative proportion of patients experiencing events over time, exhibiting a monotonically non-decreasing trend; OS curve represents the proportion of patients surviving over time, showing a monotonically non-increasing trend. OS overall survival, NRM non-relapse mortality

References

1. Lin L, Hong M, Fu X. Mycophenolate mofetil increases the risk of diarrhea in allogeneic hematopoietic stem cell transplantation recipients. J Int Med Res. 2023;51(10):3000605231206968.
2. Zhao Y, Wu H, Shi J, Luo Y, Li X, Lan J, et al. Ruxolitinib combined with etanercept induce a rapid response to corticosteroid-refractory severe acute graft vs host disease after allogeneic stem cell transplantation: Results of a multi-center prospective study. Am J Hematol. 2020;95(9):1075-84.
3. Xu Z, Mo X, Kong Y, Wen Q, Han T, Lyu M, et al. Mini-dose methotrexate combined with methylprednisolone as a first-line treatment for acute graft-versus-host disease: A phase 2 trial. J Transl Int Med. 2023;11(3):255-64.
4. Zhao K, Lin R, Fan Z, Chen X, Wang Y, Huang F, et al. Mesenchymal stromal cells plus basiliximab, calcineurin inhibitor as treatment of steroid-resistant acute graft-versus-host disease: a multicenter, randomized, phase 3, open-label trial. J Hematol Oncol. 2022;15(1):22.
5. Zeiser R, Von Bubnoff N, Butler J, Mohty M, Niederwieser D, Or R, et al. Ruxolitinib for glucocorticoid-refractory acute graft-versus-host disease. N Engl J Med. 2020;382(19):1800-10.
6. Ramirez CB, Bozdin A, Frank A, Maley W, Doria C. Optimizing use of basiliximab in liver transplantation. Transpl Res Risk Man. 2010;2:1-10.
7. Kovarik J, Breidenbach T, Gerbeau C, Korn A, Schmidt AG, Nashan B. Disposition and immunodynamics of basiliximab in liver allograft recipients. Clin Pharmacol Ther. 1998;64(1):66-72.
8. Mo XD, Hong SD, Zhao YL, Jiang EL, Chen J, Xu Y, et al. Basiliximab for steroid-refractory acute graft-versus-host disease: a real-world analysis. Am J Hematol. 2022;97(4):458-69.
9. Jagasia M, Perales MA, Schroeder MA, Ali H, Shah NN, Chen YB, et al. Ruxolitinib for the treatment of steroid-refractory acute GVHD (REACH1): a multicenter, open-label phase 2 trial. Blood. 2020;135(20):1739-49.
10. Shen MZ, Liu XX, Qiu ZY, Xu LP, Zhang XH, Wang Y, et al. Efficacy and safety of mesenchymal stem cells treatment for multidrug-resistant graft-versus-host disease after haploidentical allogeneic hematopoietic stem cell transplantation. Ther Adv Hematol. 2022;13:20406207211072838.
11. Jiang E, Qian K, Wang L, Yang D, Shao Y, Hu L, et al. Efficacy and safety of human umbilical cord-derived mesenchymal stem cells versus placebo added to second-line therapy in patients with steroid-refractory acute graft-versus-host disease: a multicentre, randomized, double-blind, phase 2 trial. BMC Med. 2024;22(1):555.
